# Supplementary material for: Is ambient air pollution associated with onset of sudden infant death syndrome: a case-crossover study in the UK
Source: BMJ Open. 2018 Apr 12;8(4):e018341. doi: 10.1136/bmjopen-2017-018341 (PMC5898297; doi:10.1136/bmjopen-2017-018341)
Supplement: Supplementary file 1 [file bmjopen-2017-018341supp001.pdf]

Conditional Poisson Regression (CPR)

Here we applied the time stratified case-crossover approach where the strata are matching days based on the same day of the week, calendar month and year that has previously been used to minimise bias [Janes et al. 2005]. That is, control days were selected from the same day of the week, within the same calendar month and year as the event day. We applied a conditional Poisson regression model which has been shown to give equivalent estimates as the conditional logistic model but with the advantage of readily allowing for overdispersion.

Thus, to fit the CPR model we first create the starta for yearXmonthXday-of-week from the original time series data and then fit the regression using the 'gnm' package adjusting for temprture spline and holday effects, conditional on starta as detailed in Armstrong et al. [2014]. An example line of R code to fit the CPR model using the R package 'gnm' is given below: *CPR_model <- gnm(sids_freq ~ Lag(pollution, k) + ns(temperature,3) + holiday, data=data, family=quasipoisson, eliminate=factor(stratum))*

where

Lag(pollution, k): pollution exposure at lag k

ns(temperature,3): temperature splines

sids_freq: SIDS count

stratum: stratum indicator (for yearXmonthXday-of-week)

References

Holly Janes, Lianne Sheppard, Thomas Lumley. Case–Crossover Analyses of Air Pollution Exposure Data, Referent Selection Strategies and Their Implications for Bias. Epidemiology 2005;16: 717-726.

Armstrong BG, Gasparrini A, Tobias A. Conditional Poisson models: a flexible alternative to conditional logistic case cross-over analysis. BMC Med Res Methodol 2014;14:122.
