# Supplementary figures and images for: Is ambient air pollution associated with onset of sudden infant death syndrome: a case-crossover study in the UK
Source: BMJ Open. 2018 Apr 12;8(4):e018341. doi: 10.1136/bmjopen-2017-018341 (PMC5898297; doi:10.1136/bmjopen-2017-018341)

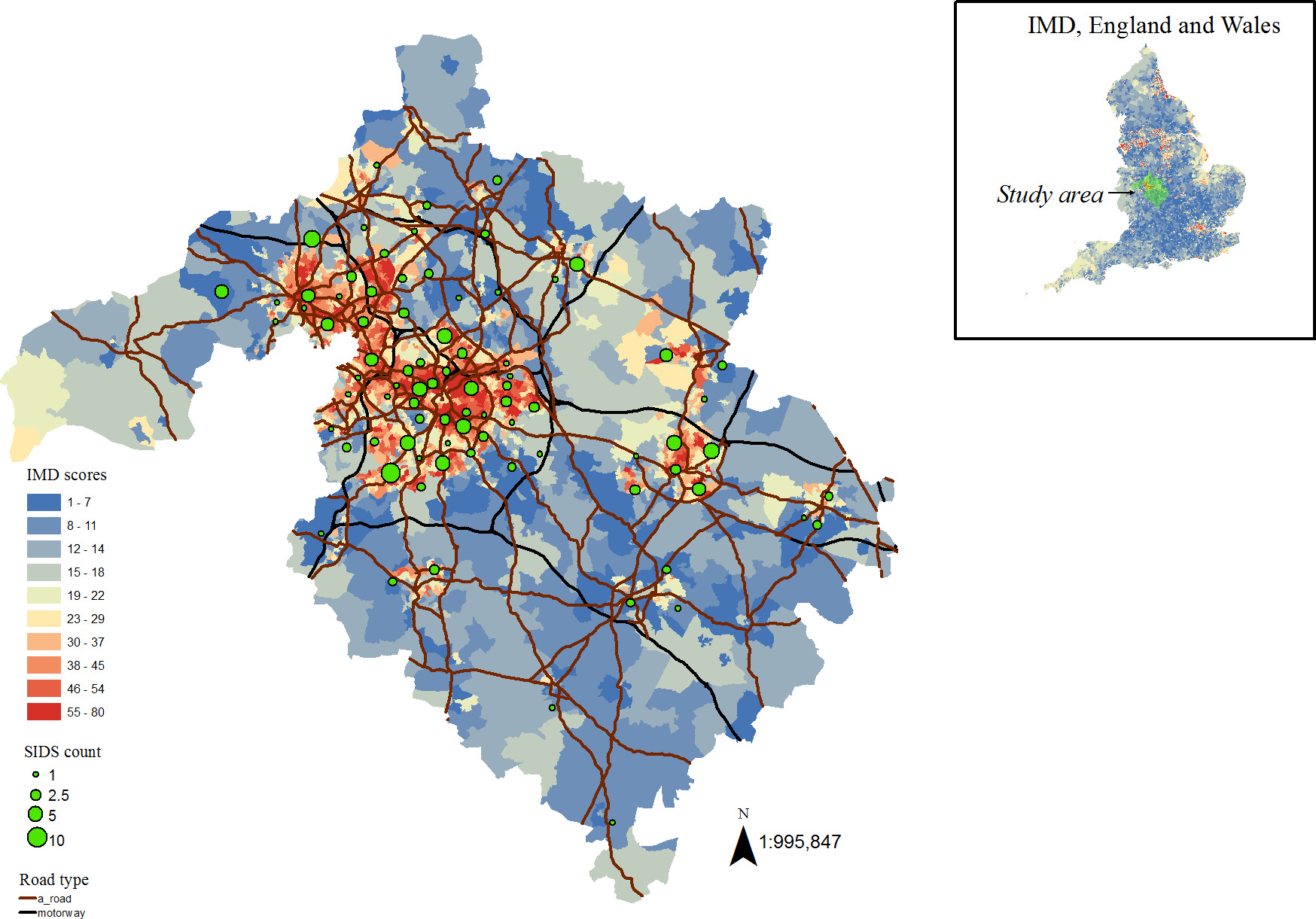

Supplement: Supplementary file 2 [file bmjopen-2017-018341supp002.jpg]

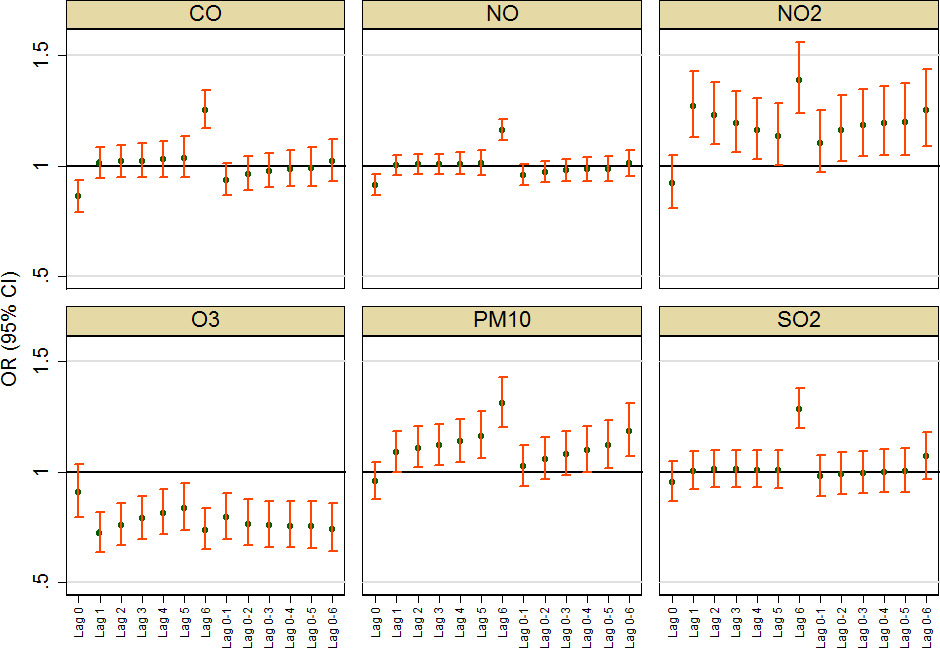

Supplement: Supplementary file 3 [file bmjopen-2017-018341supp003.jpg]
