# Supplementary material for: Is ambient air pollution associated with onset of sudden infant death syndrome: a case-crossover study in the UK
Source: BMJ Open. 2018 Apr 12;8(4):e018341. doi: 10.1136/bmjopen-2017-018341 (PMC5898297; doi:10.1136/bmjopen-2017-018341)
Supplement: Author's manuscript [file bmjopen-2017-018341.draft_revisions.pdf]

BMJ Open

BMJ Open is committed to open peer review. As part of this commitment we make the peer review history of every article we publish publicly available.

When an article is published we post the peer reviewers' comments and the authors' responses online. We also post the versions of the paper that were used during peer review. These are the versions that the peer review comments apply to.

The versions of the paper that follow are the versions that were submitted during the peer review process. They are not the versions of record or the final published versions. They should not be cited or distributed as the published version of this manuscript.

BMJ Open is an open access journal and the full, final, typeset and author-corrected version of record of the manuscript is available on our site with no access controls, subscription charges or pay-per-view fees (http://bmjopen.bmj.com).

If you have any questions on BMJ Open's open peer review process please email info.bmjopen@bmj.com

BMJ Open

Ambient Air Pollution and Sudden Infant Death Syndrome: A case crossover study

Journal:	*BMJ Open*
Manuscript ID	bmjopen-2017-018341
Article Type:	Research
Date Submitted by the Author:	21-Jun-2017
Complete List of Authors:	Litchfield, Ian; University of Birmingham, Institute of Applied Health Research<br>Ayres, Jon; University of Birmingham, Institute of Applied Health Research<br>Jaakkola, Jouni; University of Oulu, Center for Environmental and Respiratory Health Research<br>Mohammed, Nuredin; University of Birmingham, Institute of Applied Health Research
**Primary Subject Heading**:	Epidemiology
Secondary Subject Heading:	Occupational and environmental medicine, Paediatrics, Public health, Respiratory medicine
Keywords:	EPIDEMIOLOGY, Community child health < PAEDIATRICS, Cot death < PAEDIATRICS, Paediatric thoracic medicine < PAEDIATRICS, PUBLIC HEALTH

SCHOLARONE™
Manuscripts

For peer review only

Ambient Air Pollution and Sudden Infant Death Syndrome: A case crossover study

Ian J Litchfield[1*], Jon G Ayres[1], Jouni J K Jaakkola[2], and Nuredin I Mohammed[1]

Affiliations

1 Institute of Applied Health Research, College of Medical and Dental Sciences
University of Birmingham
Edgbaston, Birmingham, B15 2TT
United Kingdom

2 Center for Environmental and Respiratory Health Research
Institute of Health Sciences
POB 5000 (Aapistie 1)
University of Oulu,
90014 Oulu, Finland

***Corresponding Author**

Dr Ian J Litchfield

Address: Institute of Applied Health Research, College of Medical and Dental Sciences
University of Birmingham
Edgbaston, Birmingham, B15 2TT
United Kingdom

Email: i.litchfield@bham.ac.uk

Telephone: +44 0121 414 6006

Fax: +44 0121 414 6217

Word count: 2460

Number of figures: 4

Number of tables: 4

For peer review only

ABSTRACT

Objectives: Air pollution has been associated with increased mortality and morbidity in several studies with indications its effect could be more severe in children. This study examined the relationship between short-term variations in criteria air pollutants and occurrence of sudden infant death syndrome (SIDS). using data from the West Midlands region in the UK.

Design: We used a case-crossover study design. widely applied in air pollution studies and particularly useful for estimating the risk of a rare acute outcome associated with short-term exposure.

Setting: The study used data from the West Midlands region in the UK

Participants: We obtained daily time series data on SIDS mortality (ICD-9: 798.0 or ICD-10: R95) for the period 1996-2006 a total of 211 SIDS events.

Primary outcome measures: The association between short-term exposure to air pollution and the occurrence of SIDS events controlling for daily birth counts and average daily temperature.

Results: For an interquartile range increase in previous day pollutant concentration, the percentage increases (95% confidence interval) in SIDS were 16 (6, 28) for PM_{10}, 1 (-7, 13) for SO_2, 4 (-5, 13) for CO, -24 (-36, -5) for O_3, 13 (0.1, 28) for NO_2, and 1 (-4, 7) for NO. Results were qualitatively similar after additional control for index of multiple deprivation (IMD) scores for most of the lag structures investigated. PM_{10} and NO_2 showed relatively consistent association which persisted across different lag structures and after adjusting for co-pollutants.

Conclusions: The results indicated ambient air pollutants, particularly PM_{10} and NO_2, were associated with increased SIDS mortality. Thus, future studies are recommended to

understand possible mechanistic explanations on the role of air pollution on SIDS incidence and the ways in which we might reduce pollution exposure among infants.

Clinical trial registration: Not applicable.

Article Summary

Strengths and limitations of this study

- Sudden infant death is the leading cause of death in healthy infants between one month and one year old. The exact cause is unknown though evidence suggests a respiratory trigger may be involved.
- Our study is the first based on time series data from the UK to investigate the relationship between common air pollutants and SIDS.
- We used a case-crossover design which is widely applied in air pollution studies and particularly useful for estimating the risk of a rare acute outcome associated with short-term exposure.
- We investigated various lags and multi-pollutant models indicating delayed effects of pollutants that persisted after controlling for co-pollutants.
- Our study is limited in power due to the comparatively small number of daily SIDS events.

INTRODUCTION

The quality of ambient air is an important factor in the health of adults and children. Ambient air quality is the second largest challenge facing public health in the UK.[1] According to the World Health Organisation (WHO), over 3.7 million premature deaths per annum may be attributed to the harmful effects of ambient air. Children it would seem are more vulnerable than any other group[2] and recent studies have indicated how even low-levels of traffic-related air pollution can have a negative impact on birth outcomes and perinatal health.[3, 4] A number of studies have also suggested a link between ambient air quality and the incidence of sudden infant death syndrome (SIDS).[5-10]

Sudden infant death is the leading cause of death in healthy infants between one month and one year old,[11] and the impact on the families is notably traumatic as the death is without warning or witness.[12] The exact cause is unknown though is likely the result of a combination of factors including susceptibility and environmental stressors such as lower social status of parents,[13] environmental tobacco smoke,[13, 14] the prone position[15] and the winter season.[5, 15] The impact of tobacco smoke suggests a respiratory trigger may be involved, however the evidence of an association between SIDS and air pollution warrant further research as findings from epidemiological studies have been inconsistent.[16, 17] and few have satisfactorily explore the impact of short-term exposure on SIDs. Our study is the first based on time series data from the UK to investigate the relationship between common air pollutants and SIDS. We have collated data from a ten-year period on concentrations of air pollution and onset of SIDS within the West Midlands one of the largest and most polluted conurbations in the UK.

For peer review only

METHODS

Data collection

We combined data on SIDS events with data on total births, air pollution, air temperature and a measure of deprivation.

Data on SIDS mortality and total births

We obtained daily time series data on SIDS mortality (ICD-9: 798.0 or ICD-10: R95) for the period 1996-2006 from the Perinatal Institute (PI). This data consisted of the date of death and the first three digits of the postcode to avoid the possibility of identifying individual cases due to the rare nature of SIDS. This allowed us to explore the effects of short-term exposure. We obtained daily births data with West Midlands' postcodes from the Office of National Statistics (ONS) for the period 1996-2006.

Air pollution data

The daily time series data on air pollution were compiled from the UK air quality archive managed by Department for the Environment, Food and Rural Affairs (DEFRA). These include a total of ten monitoring stations in the West Midlands measuring PM_{10}, SO_2, NO_2, NO, NOx, CO and O_3 including sites within Birmingham, Coventry, Walsall and Wolverhampton. Before 1996 the reliability of air quality data was inconsistent. Not all monitoring stations had measurements for the range of pollutants over the entire study period and the precise pollutants measured at each monitoring centre are described in Table 1.

For peer review only - http://bmjopen.bmj.com/site/about/guidelines.xhtml

Table 1: Pollutants measured and corresponding time period by monitoring stations

Name (postcode area)	Pollutants	Time period[a]
Birmingham Centre (B)	PM_{10}, SO_2, NO_2,NO, NOx, CO, O_3	01/01/1996-31/12/2006
Birmingham East (B)	PM_{10}, SO_2, NO_2,NO, CO, O_3	01/01/1996-03/08/2004
Birmingham Tyburn (B)	PM_{10}, SO_2, NO_2,NO, CO, O_3	16/08/2004-31/12/2006
Oldbury (B)	PM_{10}, SO_2, NO_2,NO, NOx, CO, O_3	27/06/1997-20/09/1998
West Bromwich (B)	PM_{10}, SO_2, NO_2,NO, NOx, CO, O_3	04/11/1998-31/12/2006
Coventry Centre (CV)	PM_{10}, SO_2, NO_2,NO, NOx, CO, O_3	19/02/1997-31/12/2006
Coventry Memorial (CV)	PM_{10}, SO_2, NO_2,NO, NOx, CO, O_3	26/02/2001-31/12/2006
Walsall Alumwell (WS)	NO_2, NOx	01/01/1996-31/12/2006
Walsall Willenhall (WS)	NO_2	13/05/1997-31/12/2006
Wolverhampton Centre (WV)	PM_{10}, SO_2, NO_2,NO, NOx, CO, O_3	19/12/1995-31/12/2006

[a]Period with at least one pollutant being measured and taking into account missing data between start and end dates.

For peer review only

We aimed to examine the association between day-to-day variability in air pollution exposure and SIDS counts over the entire region rather than contrasting exposure and outcome between areas within the West Midlands region. Therefore the daily pollution data for West Midlands were calculated by averaging across all monitoring stations with available measurements.

Temperature data

Data on daily minimum and maximum temperature were compiled for weather monitoring stations in the West Midlands from the Meteorological Office British Atmospheric Data Centre (BADC).[18] We used the average daily temperature which was calculated by taking the average of the minimum and maximum temperature at each monitoring station for each day to obtain the daily average temperature for the entire West Midlands.

Index of multiple deprivation (IMD) score

The IMD score is a composite measure based on seven dimensions of deprivation including income deprivation, employment deprivation, health deprivation and disability, education deprivation, crime deprivation, barriers to housing and services deprivation and living environment deprivation. The data for the 2010 IMD at postcode level were downloaded from EDINA Digimap ShareGeo facility, an online spatial data repository.[19]

Statistical analysis

The case-crossover design was used to investigate the association between short-term exposure to air pollution and the occurrence of SIDS events controlling for daily birth counts and

For peer review only - http://bmjopen.bmj.com/site/about/guidelines.xhtml

average daily temperature. This design, introduced by Maclure,[20] has been widely applied in air pollution studies and is particularly useful for estimating the risk of a rare acute outcome associated with short-term exposure.[21-24] In case-crossover design each case acts as their own control and like case-control studies[25] the distribution of exposure is compared between 'cases' and 'controls'. That is, exposure at the time just prior to the event ('case' or 'index' time) is compared with a set of 'control' times that represent the expected distribution of exposure for non-event follow-up times. The design helps primarily to control for confounding by subject-specific factors which remain static over time such as ethnicity and gender.

We applied the time stratified case-crossover approach where the strata are matching days based on the same day of the week, calendar month and year. That is, control days were selected from the same day of the week, within the same calendar month and year as the event day. We applied a conditional Poisson regression model which has been shown to give equivalent estimates as the conditional logistic model but with the advantage of readily allowing for overdispersion and autocorrelation.[26] All our models assume a linear effect for air pollution reported in previous studies[27-29] while temperature effects are likely to be non-linear and were modelled using natural splines with 3 degrees of freedom.[30, 31]

Sensitivity analyses

The primary aim was to investigate the risk of SIDS events in relation to air pollution on the previous day in single pollutant models. To examine sensitivities to our *a priori* model specification, additional lag structures were explored including single lags of 0,1,2,…,6 days and also the corresponding average of lags 0-1, 0-2, 0-3,…, 0-6. Moreover, the association between

For peer review only - http://bmjopen.bmj.com/site/about/guidelines.xhtml

SIDS and air pollution was examined after adjusting for the effect of each of the pollutants PM_{10}, SO_2, NO_2, NO, CO and O_3 as a second pollutant in turn. The aim of this two-pollutant modelling approach was to account for potential confounding effect of co-pollutants. Further control for potential confounding effect of the IMD scores was made in both single and multi-pollutant models after categorising the scores into five groups (quintiles). We excluded NOx from the sensitivity analyses as it showed very strong correlation particularly with NO (r=0.96).

Results are presented as percentage increases in mortality with 95% confidence intervals (CIs) for an interquartile range (IQR) increase in pollutant concentration. Hypothesis tests were two-sided with a significance level of 0.05. All analyses were performed using the R statistical package.[32]

For peer review only

RESULTS

Descriptive statistics

Over the study period (1996–2006), there were 211 SIDS events across the four postcode areas (B, CV, WS and WV) included in the analyses which accounted for about approximately 5% of the days within the study period (Table 2). In the same period and location, a total of 943,937 live (single) births were registered.

Table 2: Average air pollution, temperature and IMD scores for selected West Midlands postcode areas with SIDS and birth counts, 1996-2006

Post town (area)	PM_{10}	SO_2	CO	O_3	NO_2	NO	NOx	Temp. (Min-Max)	IMD score	SIDS event (%)	Birth count (%)
Birmingham (B)	23.8	6.8	0.4	38.5	33.9	17.5	60	7.2-12.8	36.6	118 (55.9)	753844 (79.9)
Coventry (CV)	18.3	11.3	0.3	32.4	22.5	9.2	36.2	7.7-13.5	23.7	43 (20.4)	91393 (9.7)
Walsall (WS)					41.5		89.4	6.4-14.0	25.2	24 (11.4)	53532 (5.7)
Wolverhampton (WV)	23.8	9.3	0.5	38.9	29.5	18.9	59.5	8.4-12.3	33.4	26 (12.3)	45168 (4.8)
Total										211 (100)	943937 (100)

For peer review only

The daily average air pollution concentrations and their standard deviations are presented in Table 3 and show that average concentrations tended to be below UK air quality limits.

For peer review only - http://bmjopen.bmj.com/site/about/guidelines.xhtml

Table 3: Descriptive statistics for daily SIDS mortality counts, air pollution, temperature and birth counts for West Midlands, 1996-2006

Variable	Mean	SD	Median	IQR	Min	Max
SIDS count	0.1	0.3	0.0	0-0	0.0	2.0
Average temperature	10.2	5.4	10.2	6.2-14.5	-4.2	25.0
Max temperature	13.1	6.0	12.9	8.5-17.6	-2.1	29.6
Min temperature	7.4	5.1	7.5	3.6-11.4	-7.5	20.7
PM_{10}	23.4	11.6	20.2	15.7-28.2	4.0	128.9
SO_2	8.5	6.9	7.2	4.4-10.2	0.0	70.8
CO	0.4	0.2	0.4	0.3-0.5	0.1	3.5
O_3	38.1	18.2	38.0	25.4-50.4	1.3	115.8
NO_2	33.2	14.3	31.2	22.3-41.8	5.9	113.0
NO	16.9	23.8	9.0	5.4-17.9	0.4	314.9
NOx	64.5	49.3	51.1	35.2-76.4	7.9	569.2
Birth count[a]	289.8	33.9	295.0	266.0-314.0	183.0	387.0
IMD score	32.3	12.6	31.7	21.3-40.5	7.2	54.9

[a]Counts are for single births only

For peer review only - http://bmjopen.bmj.com/site/about/guidelines.xhtml

Table 4 shows that there was generally a strong correlation between the levels of pollutants investigated with the exception of the weaker correlation between O_3 and PM_{10} (r=-0.26) and with SO_2 (r=-0.34).

Table 4: Pairwise Pearson correlations coefficients (r) for pollutants and temperature

	PM_{10}	SO_2	CO	O_3	NO_2	NO	NOx
SO_2	0.53						
CO	0.56	0.59					
O_3	-0.26	-0.34	-0.56				
NO_2	0.66	0.56	0.76	-0.62			
NO	0.54	0.48	0.87	-0.58	0.73		
NOx	0.59	0.56	0.90	-0.62	0.86	0.96	
Temperature	-0.08	-0.24	-0.39	0.40	-0.40	-0.38	-0.40

For peer review only - http://bmjopen.bmj.com/site/about/guidelines.xhtml

Comparing the four postcode areas, Birmingham had the highest SIDS mortality (about 56%) and births rates (about 80%). Air pollution concentrations were more or less similar for Birmingham and Wolverhampton except slightly lower levels of NO_2 and higher levels of SO_2 observed for the latter. Coventry had the lowest pollution level with respect to all pollutants except SO_2. Walsall had measurements for NO_2 and NOx only which were very large compared to the other postcode areas (Table 2). Similarly, Birmingham and Wolverhampton had higher average deprivation scores compared to Coventry and Walsall (Table 2). Also, SIDS counts and pollution levels tended to be larger in highly deprived areas and near motorways and A-road networks which may be a reflection of population density (Figure 1; see Supplementary File: Figure S1).

Looking at the temporal distribution of SIDS occurrence over the study period, the highest proportions of deaths were recorded in 1997 (about 14%), 1999 (about 13%) and 2003 (about 12%). There was also indication of seasonal pattern for SIDS occurrence; the highest proportions of SIDS were observed in January and February (about 10%) and among the relatively colder months and in July (about 14%) among the warmer months (see Supplementary File: Table S1).

Case-crossover analysis

Figure 2 shows the estimated odds ratio (OR) (95% CI) for the association of SIDS events with each of the pollutants considered based on the conditional Poisson model. For an interquartile range increase in previous day pollutant concentration, the percentage increase (95% confidence interval) for the risk of SIDS death was 16 (6, 28); $p = 0.001$ for PM_{10}, 1 (-7, 13); $p = 0.84$ for SO_2, 4 (-5, 13) ; $p = 0.43$ for CO, -24 (-36, -5) ; $p = 0.01$ for O_3, 13 (0.1, 28)

; p = 0.048 for NO_2, 1 (-4, 7) ; p = 0.65 for NO and 7 (-2, 17) ; p = 0.12 for NOx. Therefore, considering pollutant levels at lag 1, significant association with increased risk of SIDS was indicated only for PM_{10} and NO_2 while a protective effect was apparent for O_3 before controlling for IMD. These results were similar at least qualitatively after adjusting for IMD but with decreased OR for PM_{10} and increased OR for NO_2.

Sensitivity analyses

Lag structures: Analyses exploring relationships between SIDS and air pollution for other lag structures (lags 0, 2, 3,…, 6 and corresponding average of lags 0-1, 0-2, 0-3,…, 0-6) indicated associations were somewhat sensitive to the choice of lag (Figure 3). In the single lag models, there was an impression of stronger delayed effects for CO, NO_2, O_3, PM_{10} and SO_2 compared to relatively recent lags (lag 0 and lag 1). The effect of NO appeared to remain comparatively flat across lags. Relatively more consistent association across lags was observed for NO_2 and PM_{10} and to some extent for CO. Likewise, in average lag models, ORs tended to increase with averaging over more lags with exception of NO where such an effect was not observed. However, only PM_{10}, NO_2 and O_3 showed association across several lags after additional control for IMD (see Supplementary File: Figure S2).

Multi-pollutant models: Further investigation using two-pollutant models also showed associations were sensitive to control of other pollutants. In general, adjusting for co-pollutants appeared to attenuate ORs towards the null except for NO_2 and PM_{10} where effects remained to persist (Figure 4). Interestingly, after controlling for PM_{10}, the protective effect of O_3 and the adverse effect of NO_2 observed in the single pollutant models were not apparent for most of the

lag choices. Similarly, none of the other pollutants showed marked association with SIDS after controlling for PM_{10} effects. The estimates from the multi-pollutant model should, however, be interpreted with caution as most of the pollutants were correlated.

For peer review only - http://bmjopen.bmj.com/site/about/guidelines.xhtml

COMMENTS

Summary of findings

In this study we hypothesised a delayed effect (lag 1) of air pollution on SIDS incidence and investigated additional lags in sensitivity analyses both before and after controlling for IMD and in single and multi-pollutant models. We found evidence suggesting association of SIDS mortality with PM_{10} and NO_2 exposure. Compared to other pollutants, their effects persisted after controlling for IMD, in multi-pollutant models and across the various lag structures investigated.

Though controlling for O_3 and IMD in the same model did attenuate the risk estimates observed for both PM_{10} and NO_2. There were no consistent associations observed with exposure to the remaining pollutants investigated (CO, SO_2, NO, NOx). An exception was the protective effect observed in relation to O_3 exposure.

Strengths and limitations

Previously studies had focused on relatively longer-term exposure to air pollutants.[33-35] Our study is one of the first to show the potential adverse effects of relatively short-term exposure to air pollution on SIDS occurrence. One of its key strengths is the investigation of various lags and multi-pollutant models indicating delayed effects of pollutants that persisted after controlling for co-pollutants. We acknowledge that our case-crossover study is limited in power due to the small number of daily SIDS events. Other epidemiological studies use data from ambient monitoring stations have shown a good level of correlation between daily personal exposure and daily ambient air pollution measurements,[36, 37] though there is the potential for measurement error that can attenuate regression coefficient estimates.

For peer review only - http://bmjopen.bmj.com/site/about/guidelines.xhtml

Detailed discussion

Previous studies reported mixed results on the relationship between SIDS and air pollution. An earlier study using visibility as a surrogate for the level of air pollution in Taiwan reported adjusted rate ratio (ARR) of 3.8 (2.8–5.1) on the day of SIDS event comparing lowest versus highest visibility. The ARR increased to 5.1 (3.2–8.1) for visibility comparison during 9 days before SIDS occurrence.[5] Later studies with actual air pollution measurements also reported increased risk for SIDS mortality but with much reduced effect sizes. Woodruff et al. and Lipfert et al. found ORs (95% CI) of 1.12 (1.07–1.17) and 1.15 (1.07–1.24) respectively per 10 $\mu g/m^3$ increase in PM_{10}.[6, 38] A multi-city study from Canada reported about 17.7% increase in SIDS occurrence for an interquartile range increase in SO_2 (lag 0) and NO_2 (lag 2) that persisted in more rigorous models adjusting for weather and seasonality. They did not find associations for O_3 and particulate pollution and the effect of CO was not robust to models choice;[8] Ritz et al. had also found such a negative association for O_3 reporting ORs (95% CI) of 0.89 (0.82–0.96) and 0.93 (0.84–1.02) for single and multi-pollutant models respectively.[33] Similar to our results they highlighted that OR estimates were attenuated in multi-pollutant models. One possible reason for the negative association of O_3 might be its negative correlation with other pollutants and it could also be confounded by season. For example, Woodruff et al. analysed O_3 effect for different seasons and found adverse O_3 effects in the summer season only[34] while Samet et al. reported similar effect for the summer but protective effect in the winter season.[39] However, Sarnat et al. argued that the negative association observed for O_3 can be explained by its negative correlation with fine particulate matter pollution.[40]

For peer review only - http://bmjopen.bmj.com/site/about/guidelines.xhtml

Conclusion

In conclusion, our findings suggest that air pollution exposure may increase risk of SIDS mortality. Thus, future studies are recommended to help understand possible mechanistic explanations on the role of air pollution on SIDS incidence and the ways in which we can reduce pollution exposure among infants.

For peer review only

For peer review only - http://bmjopen.bmj.com/site/about/guidelines.xhtml

ACKNOWLEDGMENTS

Funding: This work was supported by The Lullaby Trust, grant number 260.

Competing interests: All authors declare: grants from The Lullaby Trust; no financial relationships with any organisations that might have an interest in the submitted work in the previous three years and no other relationships or activities that could appear to have influenced the submitted work.

Author contributions statement: NIM contributed to the research design, performed the data management, data analysis and interpretation, and drafted the initial manuscript; JGA contributed to the conceptualisation of the study, data interpretation and critically reviewed the manuscript; JJKJ contributed to the conceptualisation of the study, data interpretation and critically reviewed the manuscript; IJL contributed to the conceptualisation of the study, initial draft manuscript and supervised the research project at all stages. All authors approved the final manuscript as submitted.

Transparency: As lead author, I can confirm that the manuscript is an honest, accurate, and transparent account of the study being reported. No important aspects have been omitted and there are no discrepancies from the planned study.

Ethics committee approval: Ethical approval was given by the University of Birmingham, Life and Health Sciences Ethical Review Committee.

For peer review only

Data sharing: No additional data available.

For peer review only

For peer review only - http://bmjopen.bmj.com/site/about/guidelines.xhtml

REFERENCES

1. Royal College of Physicians, Royal College of Paediatrics and Child Health. Every breath we take: The lifelong impact of air pollution. 2016. https://www.rcplondon.ac.uk/projects/outputs/every-breath-we-take-lifelong-impact-air-pollution (accessed Apr 2017).
2. World Health Organization. Burden of disease from Ambient Air Pollution for 2012. 2014. http://www.who.int/phe/health_topics/outdoorair/databases/AAP_BoD_results_March2014.pdf?ua=1 (accessed Apr 2017).
3. Brauer M, Lencar C, Tamburic L, et al. A cohort study of traffic-related air pollution impacts on birth outcomes. Environ Health Perspect 2008;116(5):680-6.
4. Fleischer NL, Merialdi M, van Donkelaar A, et al. Outdoor air pollution, preterm birth, and low birth weight: analysis of the World Health Organization global survey on maternal and perinatal health. Environ Health Perspect 2014;122(4):425-30.
5. Knobel HH, Chen CJ, Liang KY. Sudden infant death syndrome in relation to weather and optimetrically measured air pollution in Taiwan. Pediatrics 1995;96(6):1106-10.
6. Lipfert FW, Zhang J, Wyzga RE. Infant mortality and air pollution: a comprehensive analysis of U.S. data for 1990. J Air Waste Manag Assoc 2000;50(8):1350-66.
7. Hunt CE. Sudden infant death syndrome and other causes of infant mortality: diagnosis, mechanisms, and risk for recurrence in siblings. Am J Respir Crit Care Med 2001;164(3):346-57.
8. Dales R, Burnett RT, Smith-Doiron M, et al. Air pollution and sudden infant death syndrome. Pediatrics 2004;113(6):e628-31.

For peer review only - http://bmjopen.bmj.com/site/about/guidelines.xhtml

9. Kaiser R, Romieu I, Medina S, et al. Air pollution attributable postneonatal infant mortality in U.S. metropolitan areas: a risk assessment study. Environ Health 2004;3(1):4.

10. Heinrich J, Slama R. Fine particles, a major threat to children. Int J Hyg Environ Health 2007;210(5):617-22.

11. National Institute of Child Health and Human Development. How many infants die from SIDS or are at risk for SIDS? 2013. https://www.nichd.nih.gov/health/topics/sids/conditioninfo/pages/risk.aspx (accessed Apr 2017).

12. Kinney HC, Thach BT. The sudden infant death syndrome. N Engl J Med 2009;361(8):795-805.

13. Leach CE, Blair PS, Fleming PJ, et al. Epidemiology of SIDS and explained sudden infant deaths. CESDI SUDI Research Group. Pediatrics 1999;104(4):e43.

14. Brooke H, Gibson A, Tappin D, et al. Case-control study of sudden infant death syndrome in Scotland, 1992-5. BMJ 1997;314(7093):1516-20.

15. Douglas AS, Allan TM, Helms PJ. Seasonality and the Sudden Infant Death Syndrome during 1987-9 and 1991-3 in Australia and Britain. BMJ 1996;312(7043):1381-3.

16. Glinianaia SV, Rankin J, Bell R, et al. Does particulate air pollution contribute to infant death? A systematic review. Environ Health Perspect 2004;112(14):1365-70.

17. Tong S, Colditz P. Air pollution and sudden infant death syndrome: a literature review. Paediatr Perinat Epidemiol 2004;18(5):327-35.

18. National Centre for Atmospheric Science, Natural Environment Research Council. British Atmospheric Data Centre. http://badc.nerc.ac.uk/home/index_beacon.html (accessed Apr 2017).

For peer review only

For peer review only - http://bmjopen.bmj.com/site/about/guidelines.xhtml

19. Pope A. "Indices of Deprivation England and Wales 2010" [ESRI Shapefile geospatial data]. 2013. https://www.sharegeo.ac.uk/handle/10672/481 (accessed Dec 2016).

20. Maclure M. The case-crossover design: a method for studying transient effects on the risk of acute events. Am J Epidemiol 1991;133(2):144-53.

21. Lee JT, Schwartz J. Reanalysis of the effects of air pollution on daily mortality in Seoul, Korea: A case-crossover design. Environ Health Perspect 1999;107(8):633-6.

22. Jaakkola JJ. Case-crossover design in air pollution epidemiology. Eur Respir J Suppl 2003;40:81s-5s.

23. Pope CA 3rd, Muhlestein JB, May HT, et al. Ischemic heart disease events triggered by short-term exposure to fine particulate air pollution. Circulation 2006;114(23):2443-8.

24. Bhaskaran K, Hajat S, Armstrong B, et al. The effects of hourly differences in air pollution on the risk of myocardial infarction: case crossover analysis of the MINAP database. BMJ 2011;343:d5531.

25. Breslow NE, Day NE. Statistical methods in cancer research. Volume I - The analysis of case-control studies. IARC Sci Publ 1980;32:5-338.

26. Armstrong BG, Gasparrini A, Tobias A. Conditional Poisson models: a flexible alternative to conditional logistic case cross-over analysis. BMC Med Res Methodol 2014;14:122.

27. Le Tertre A, Medina S, Samoli E, et al. Short-term effects of particulate air pollution on cardiovascular diseases in eight European cities. J Epidemiol Community Health 2002;56(10):773-9.

28. Katsouyanni K, Samet JM, Anderson HR, et al. Air pollution and health: a European and North American approach (APHENA). Res Rep Health Eff Inst 2009;142:5-90.

For peer review only

29. Scheers H, Mwalili SM, Faes C, et al. Does air pollution trigger infant mortality in Western Europe? A case-crossover study. Environ Health Perspect 2011;119(7):1017-22.

30. Braga AL, Zanobetti A, Schwartz J. The effect of weather on respiratory and cardiovascular deaths in 12 U.S. cities. Environ Health Perspect 2002;110(9):859-63.

31. Armstrong B. Models for the relationship between ambient temperature and daily mortality. Epidemiology 2006;17(6):624-31.

32. R Core Team. R: A Language and Environment for Statistical Computing. 2013. http://www.R-project.org (accessed Apr 2017).

33. Ritz B, Wilhelm M, Zhao Y. Air pollution and infant death in southern California, 1989-2000. Pediatrics 2006;118(2):493-502.

34. Woodruff TJ, Darrow LA, Parker JD. Air pollution and postneonatal infant mortality in the United States, 1999-2002. Environ Health Perspect 2008;116(1):110-5.

35. Woodruff TJ, Parker JD, Schoendorf KC. Fine Particulate Matter (PM(2.5)) Air Pollution and Selected Causes of Postneonatal Infant Mortality in California. Environ Health Perspect 2006;114(5):786-90.

36. Janssen NA, Lanki T, Hoek G, et al. Associations between ambient, personal, and indoor exposure to fine particulate matter constituents in Dutch and Finnish panels of cardiovascular patients. Occup Environ Med 2005;62(12):868-77.

37. Williams R, Suggs J, Zweidinger R, et al. The 1998 Baltimore Particulate Matter Epidemiology-Exposure Study: part 1. Comparison of ambient, residential outdoor, indoor and apartment particulate matter monitoring. J Expo Anal Environ Epidemiol 2000;10:518-32.

For peer review only

For peer review only - http://bmjopen.bmj.com/site/about/guidelines.xhtml

38. Woodruff TJ, Grillo J, Schoendorf KC. The relationship between selected causes of postneonatal infant mortality and particulate air pollution in the United States. Environ Health Perspect 1997;105(6):608-12.

39. Samet JM, Dominici F, Curriero FC, et al. Fine particulate air pollution and mortality in 20 U.S. cities, 1987–1994. N Engl J Med 2000;343(24):1742-9.

40. Sarnat J, Schwartz J, Suh H. Comment on: Fine particulate air pollution and mortality in 20 U.S. cities. N Engl J Med 2001;344(16):1253-4.

FIGURE LEGENDS

Fig. 1 Air pollution monitoring sites with road networks (motorways and A-roads) and SIDS events in the study area. We used ArcGIS Desktop 10.2 (http://www.esri.com/software/arcgis) to create the map combining shapefiles for postcode areas and motorways and overlaying air pollution and SIDS data.

Fig. 2 Estimated risk of SIDS for an IQR increase in lag 1 pollutant concentration before and after controlling for quintiles of IMD scores

Fig. 3 Risk of SIDS associated with an IQR increase in pollution for selected lags and average of lags

Fig. 4 Risk of SIDS associated with an IQR increase in air pollution after controlling for co-pollutants (panel titles indicate: main and co-pollutant respectively)

For peer review only - http://bmjopen.bmj.com/site/about/guidelines.xhtml

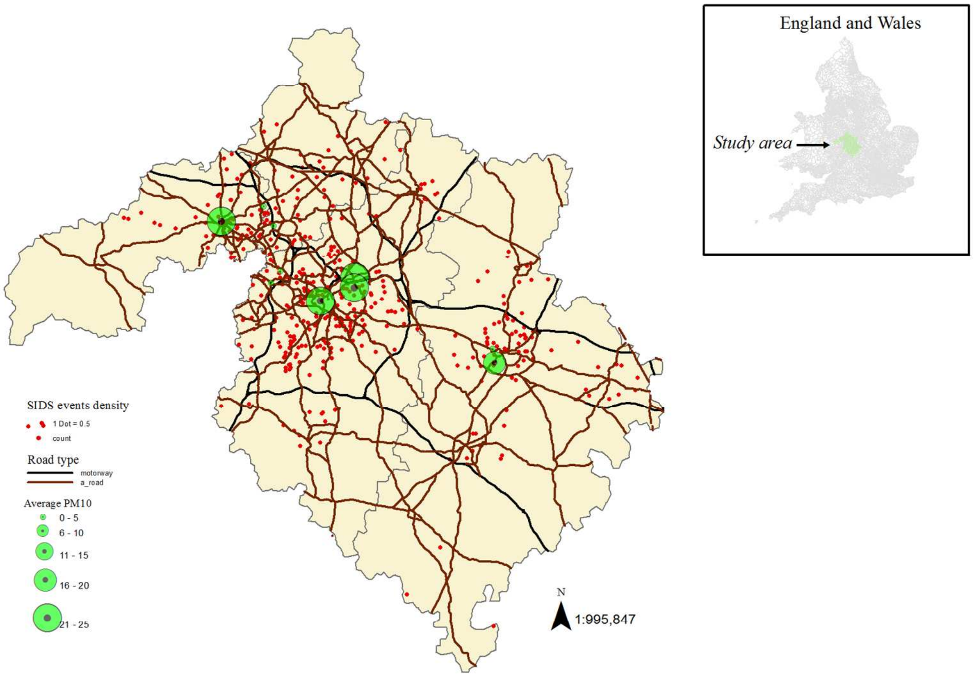

Air pollution monitoring sites with road networks (motorways and A-roads) and SIDS events in the study area. We used ArcGIS Desktop 10.2 (http://www.esri.com/software/arcgis) to create the map combining shapefiles for postcode areas and motorways and overlaying air pollution and SIDS data.

152x114mm (300 x 300 DPI)

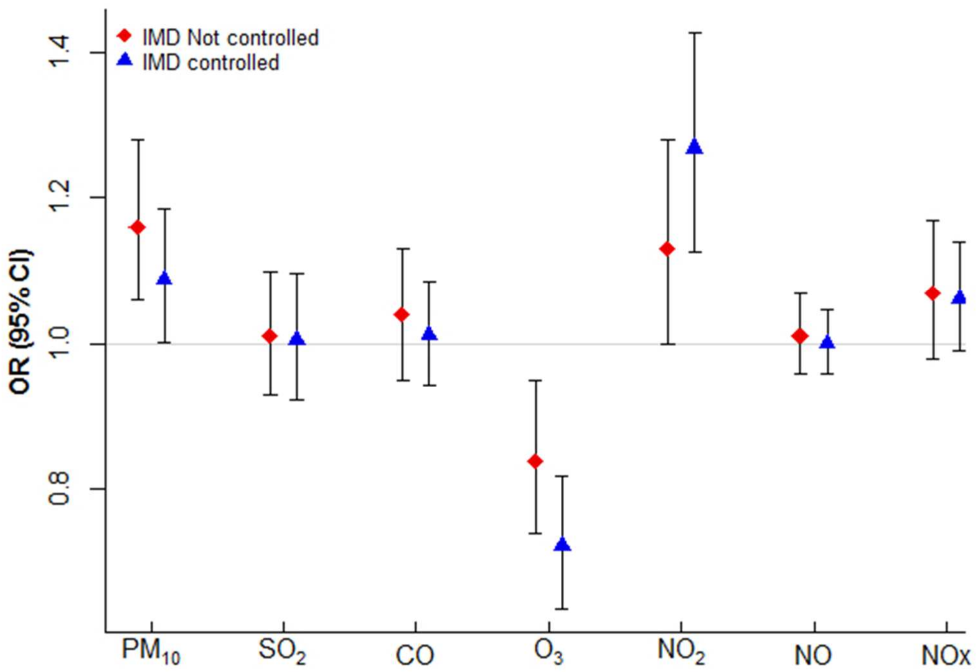

Estimated risk of SIDS for an IQR increase in lag 1 pollutant concentration before and after controlling for quintiles of IMD scores

160x109mm (96 x 96 DPI)

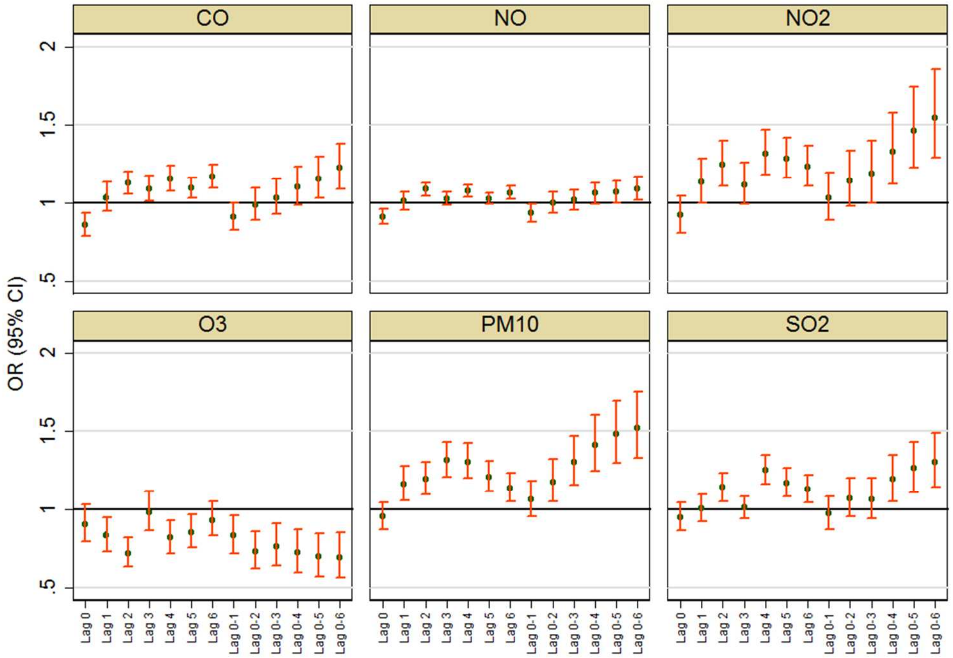

Risk of SIDS associated with an IQR increase in pollution for selected lags and average of lags

352x256mm (72 x 72 DPI)

ew only

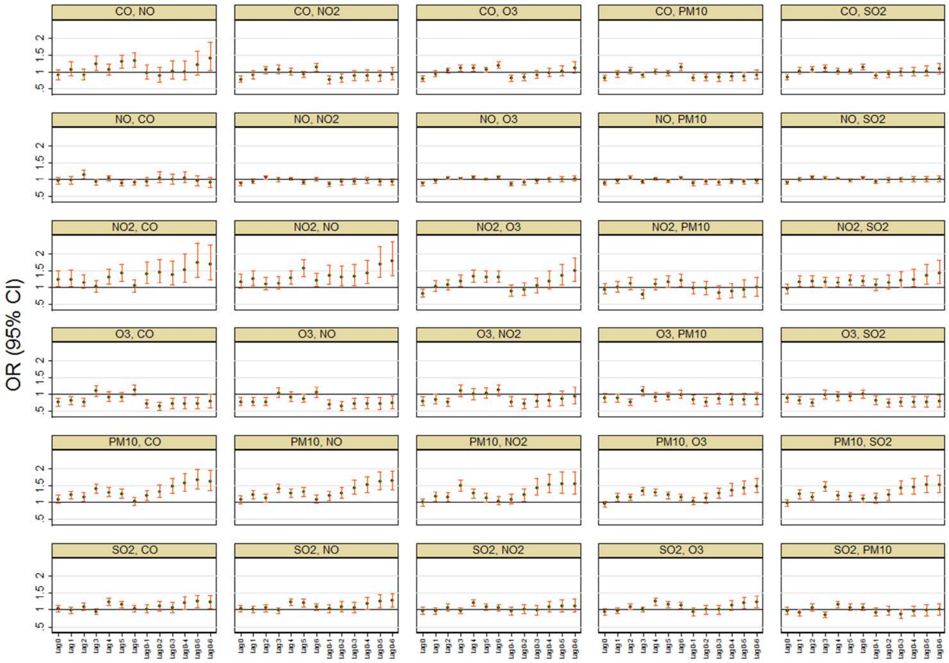

Risk of SIDS associated with an IQR increase in air pollution after controlling for co-pollutants (panel titles indicate: main and co-pollutant respectively)

438x319mm (72 x 72 DPI)

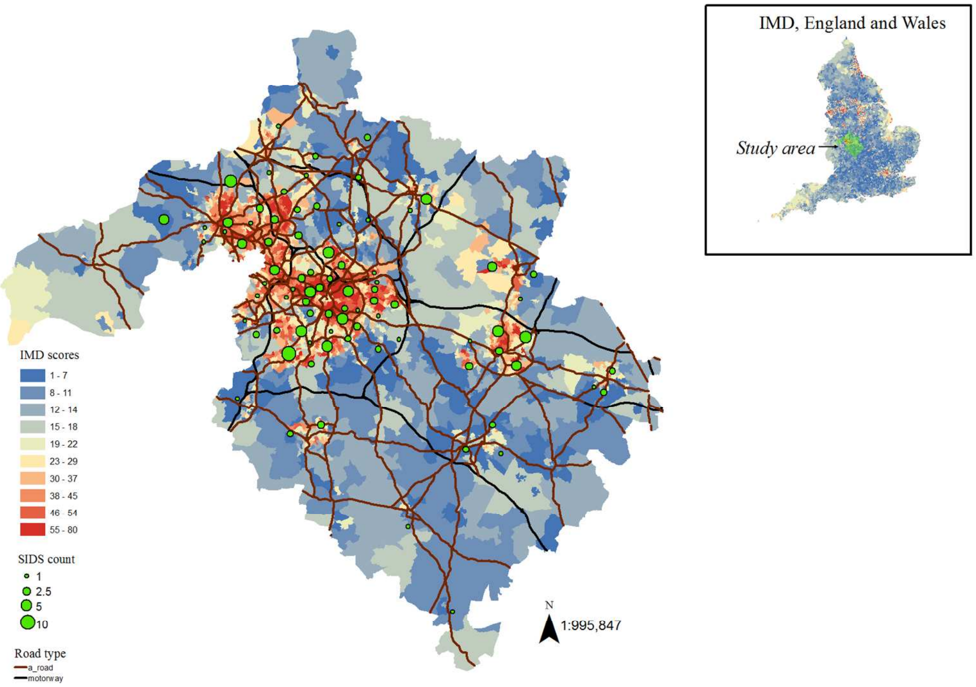

152x114mm (300 x 300 DPI)

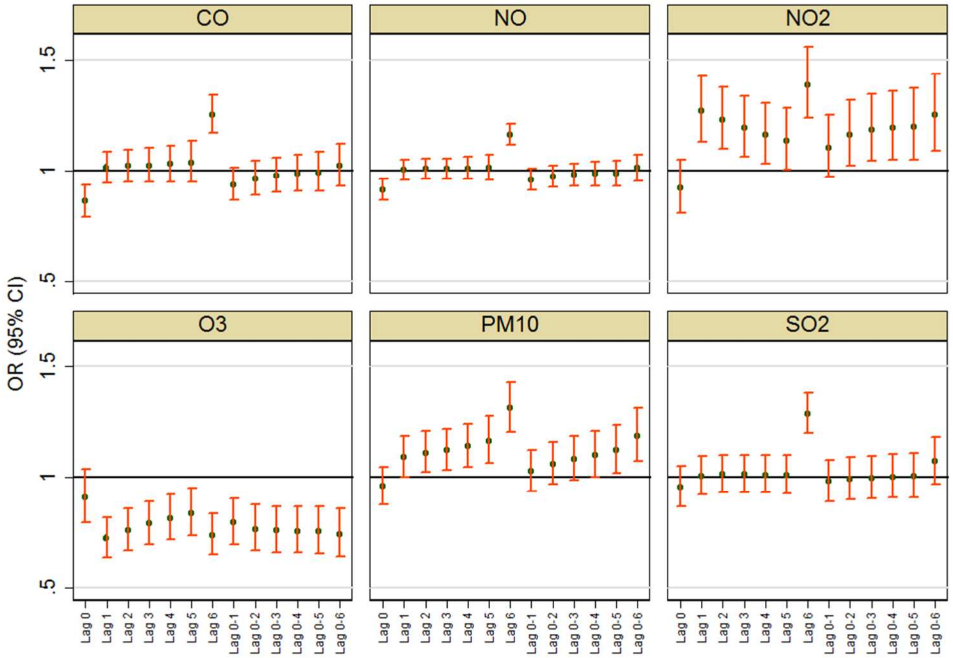

352x256mm (72 x 72 DPI)

STROBE Statement—checklist of items that should be included in reports of observational studies

	Item No	Recommendation
**Title and abstract**	1	Ambient Air Pollution and Sudden Infant Death Syndrome: a case crossover study
		Page 2
**Introduction**		
Background/rationale	2	Page 4
Objectives	3	Page 4
**Methods**		
Study design	4	Page 5
Setting	5	Page 5
Participants	6	Page 5
Variables	7	Page 6
Data sources/ measurement	8*	Page 5-7
Bias	9	Page 5-7
Study size	10	Page 5
Quantitative variables	11	Page 7
Statistical methods	12	(*a*) Describe all statistical methods, including those used to control for confounding Page 5-7
		(*b*) Describe any methods used to examine subgroups and interactions Page 7
		(*c*) Explain how missing data were addressed Page 5
		(*e*) Describe any sensitivity analyses Page 8

Continued on next page

For peer review only

For peer review only - http://bmjopen.bmj.com/site/about/guidelines.xhtml

**Results**		
Participants	13*	(a) Report numbers of individuals at each stage of study—eg numbers potentially eligible, examined for eligibility, confirmed eligible, included in the study, completing follow-up, and analysed<br>Page 9
Descriptive data	14*	(a) Give characteristics of study participants (eg demographic, clinical, social) and information on exposures and potential confounders<br>Page 9
Outcome data	15*	Page 10-11
Main results	16	Page 10-11
Other analyses	17	Page 12-13
**Discussion**		
Key results	18	Page 14
Limitations	19	Page 14
Interpretation	20	Page 15
Generalisability	21	Page 16
**Other information**		
Funding	22	The Lullaby Trust

*Give information separately for cases and controls in case-control studies and, if applicable, for exposed and unexposed groups in cohort and cross-sectional studies.

Note: An Explanation and Elaboration article discusses each checklist item and gives methodological background and published examples of transparent reporting. The STROBE checklist is best used in conjunction with this article (freely available on the Web sites of PLoS Medicine at http://www.plosmedicine.org/, Annals of Internal Medicine at http://www.annals.org/, and Epidemiology at http://www.epidem.com/). Information on the STROBE Initiative is available at www.strobe-statement.org.

For peer review only

BMJ Open

Is Ambient Air Pollution associated with onset of Sudden Infant Death Syndrome: A case crossover study in the UK

Journal:	*BMJ Open*
Manuscript ID	bmjopen-2017-018341.R1
Article Type:	Research
Date Submitted by the Author:	20-Dec-2017
Complete List of Authors:	Litchfield, Ian; University of Birmingham, Institute of Applied Health Research<br>Ayres, Jon; University of Birmingham, Institute of Applied Health Research<br>Jaakkola, Jouni; University of Oulu, Center for Environmental and Respiratory Health Research<br>Mohammed, Nuredin; University of Birmingham, Institute of Applied Health Research
**Primary Subject Heading**:	Epidemiology
Secondary Subject Heading:	Occupational and environmental medicine, Paediatrics, Public health, Respiratory medicine
Keywords:	EPIDEMIOLOGY, Community child health < PAEDIATRICS, Cot death < PAEDIATRICS, Paediatric thoracic medicine < PAEDIATRICS, PUBLIC HEALTH

SCHOLARONE™ Manuscripts

only

Is Ambient Air Pollution associated with onset of Sudden Infant Death Syndrome: A case crossover study in the UK

Ian J Litchfield[1*], Jon G Ayres[1], Jouni J K Jaakkola[2], and Nuredin I Mohammed[1,3]

Affiliations

1 Institute of Applied Health Research, College of Medical and Dental Sciences

University of Birmingham

Edgbaston, Birmingham, B15 2TT

United Kingdom

2 Center for Environmental and Respiratory Health Research

Faculty of Medicine

POB 5000 (Aapistie 5B)

90014 Oulu, Finland

3 Medical Research Council Unit The Gambia

Atlantic Road

PO Box 273, Banjul

The Gambia

***Corresponding Author**

Dr Ian J Litchfield

Address: Institute of Applied Health Research, College of Medical and Dental Sciences

University of Birmingham

Edgbaston, Birmingham, B15 2TT

United Kingdom

Email: i.litchfield@bham.ac.uk

Telephone: +44 0121 414 6006

Fax: +44 0121 414 6217

For peer review only

Word count: 3024

Number of figures: 4

Number of tables: 4

For peer review only

For peer review only - http://bmjopen.bmj.com/site/about/guidelines.xhtml

ABSTRACT

Objectives: Air pollution has been associated with increased mortality and morbidity in several studies with indications its effect could be more severe in children. This study examined the relationship between short-term variations in criteria air pollutants and occurrence of sudden infant death syndrome (SIDS).

Design: We used a case-crossover study design which is widely applied in air pollution studies and particularly useful for estimating the risk of a rare acute outcome associated with short-term exposure.

Setting: The study used data from the West Midlands region in the UK.

Participants: We obtained daily time series data on SIDS mortality (ICD-9: 798.0 or ICD-10: R95) for the period 1996-2006 with a total of 211 SIDS events.

Primary outcome measures: Daily counts of SIDS events.

Results: For an interquartile range increase in previous day pollutant concentration, the percentage increases (95% confidence interval) in SIDS were 16 (6, 27) for PM_{10}, 1 (-7, 10) for SO_2, 5 (-4, 14) for CO, -17 (-27, -6) for O_3, 16 (2, 31) for NO_2, and 2 (-3, 8) for NO after controlling for average temperature and national holidays. PM_{10} and NO_2 showed relatively consistent association which persisted across different lag structures and after adjusting for co-pollutants.

Conclusions: The results indicated ambient air pollutants, particularly PM_{10} and NO_2, were associated with increased SIDS mortality. Thus, future studies are recommended to understand possible mechanistic explanations on the role of air pollution on SIDS incidence and the ways in which we might reduce pollution exposure among infants.

Clinical trial registration: Not applicable.

Keywords: Air pollution; Child health; Time-series; Infant mortality.

Article Summary

Strengths and limitations of this study

- Sudden infant death is the leading cause of death in healthy infants between one month and one year olds and our study is the first based on time series data from the UK to investigate the relationship between common air pollutants and SIDS.
- Based on a case-crossover design, we found associations with onset of SIDS and concentration of PM_{10} and NO_2 that lasted days after the initial exposure.
- Though our study is limited in power due to the comparatively small number of daily SIDS events our chosen design is widely applied in air pollution studies and particularly useful for estimating the risk of a rare acute outcome associated with short-term exposure.

INTRODUCTION

The quality of ambient air is an important factor in the health of adults and children. Ambient air quality is the second largest challenge facing public health in the UK.[1] According

to the World Health Organisation (WHO), over 3.7 million premature deaths per annum may be attributed to the harmful effects of ambient air. Children it would seem are more vulnerable than any other group[2] and recent studies have indicated how even low-levels of traffic-related air pollution can have a negative impact on birth outcomes and perinatal health.[3, 4] A number of studies have also suggested a link between ambient air quality and the incidence of sudden infant death syndrome (SIDS).[5-12] and here we examine the effects of the short term variations in air pollution and the onset of SIDS.

Sudden infant death is the leading cause of death in healthy infants between one month and one year old,[13] and the impact on the families is notably traumatic as the death is without warning or witness.[14] The exact cause continues to tax researchers [15] though is likely the result of a combination of factors including susceptibility and environmental stressors such as lower social status of parents,[16] environmental tobacco smoke,[16, 17] the prone position[18] and the winter season.[5, 18] The impact of tobacco smoke suggests a respiratory trigger may be involved, however the evidence of an association between SIDS and air pollution warrant further research as findings from epidemiological studies have been inconsistent.[19, 20] and few have satisfactorily explored the impact of short-term exposure on SIDs. Our study is the first based on time series data from the UK to investigate the relationship between common air pollutants and SIDS. We have collated data from a ten-year period on concentrations of air pollution and onset of SIDS within the West Midlands one of the largest and most polluted conurbations in the UK and have conducted a case-crossover study to determine any associations.

For peer review only

METHODS

Settings

The West Midlands is a metropolitan county in the centre of the UK. It has a population of some 2.8 million. [21] The West Midlands is one of the most heavily urbanized counties in the UK and forms the most populated conurbation in the UK outside London, It is at the heart of the UK motorway network and remains a significant centre of the UK's manufacturing industry.

Data collection

We combined data on SIDS events with data on total births, air pollution, air temperature and a measure of deprivation.

Data on SIDS mortality and total births

We obtained daily time series data on SIDS mortality (ICD-9: 798.0 or ICD-10: R95) for the period 1996-2006 from the Perinatal Institute (PI). All cases were between 0 and 12 months old at onset. This data consisted of the date of death and the first three digits of the postcode to avoid the possibility of identifying individual cases due to the rare nature of SIDS. This allowed us to explore the effects of short-term exposure. We obtained daily births data with West Midlands' postcodes from the Office of National Statistics (ONS) for the period 1996-2006 and was used for descriptive analysis only.

Air pollution data

The daily time series data on air pollution were compiled from the UK air quality archive managed by Department for the Environment, Food and Rural Affairs (DEFRA). These include a total of ten monitoring stations in the West Midlands measuring PM_{10}, SO_2, NO_2, NO, NOx, CO and O_3 including sites within Birmingham, Coventry, Walsall and Wolverhampton. Before 1996 the reliability of air quality data was inconsistent. Not all monitoring stations had measurements for the range of pollutants over the entire study period and the precise pollutants measured at each monitoring centre are described in Table 1.

Table 1: Pollutants measured and corresponding time period by monitoring stations

Name (postcode area)	Pollutants	Time period[a]
Birmingham Centre (B)	PM_{10}, SO_2, NO_2,NO, NOx, CO, O_3	01/01/1996-31/12/2006
Birmingham East (B)	PM_{10}, SO_2, NO_2,NO, CO, O_3	01/01/1996-03/08/2004
Birmingham Tyburn (B)	PM_{10}, SO_2, NO_2,NO, CO, O_3	16/08/2004-31/12/2006
Oldbury (B)	PM_{10}, SO_2, NO_2,NO, NOx, CO, O_3	27/06/1997-20/09/1998
West Bromwich (B)	PM_{10}, SO_2, NO_2,NO, NOx, CO, O_3	04/11/1998-31/12/2006
Coventry Centre (CV)	PM_{10}, SO_2, NO_2,NO, NOx, CO, O_3	19/02/1997-31/12/2006
Coventry Memorial (CV)	PM_{10}, SO_2, NO_2,NO, NOx, CO, O_3	26/02/2001-31/12/2006
Walsall Alumwell (WS)	NO_2, NOx	01/01/1996-31/12/2006
Walsall Willenhall (WS)	NO_2	13/05/1997-31/12/2006
Wolverhampton Centre (WV)	PM_{10}, SO_2, NO_2,NO, NOx, CO, O_3	19/12/1995-31/12/2006

[a]Period with at least one pollutant being measured and taking into account missing data between start and end dates.

For peer review only - http://bmjopen.bmj.com/site/about/guidelines.xhtml

We aimed to examine the association between day-to-day variability in air pollution exposure and SIDS counts over the entire region rather than contrasting exposure and outcome between areas within the West Midlands region. Therefore the daily pollution data for West Midlands were calculated by averaging across all monitoring stations with available measurements as our models were based on the temporal relationship between air pollution and SIDS; we did not fit a spatial model.

Temperature data

Data on daily minimum and maximum temperature were compiled for weather monitoring stations in the West Midlands from the Meteorological Office British Atmospheric Data Centre (BADC).[22] We used the average daily temperature which was calculated by taking the average of the minimum and maximum temperature at each monitoring station for each day to obtain the daily average temperature for the entire West Midlands.

Index of multiple deprivation (IMD) score

The IMD score is a composite measure based on seven dimensions of deprivation including income deprivation, employment deprivation, health deprivation and disability, education deprivation, crime deprivation, barriers to housing and services deprivation and living environment deprivation. The data for the 2010 IMD at postcode level were downloaded from EDINA Digimap ShareGeo facility, an online spatial data repository and was used for descriptive analysis only.[23]

For peer review only - http://bmjopen.bmj.com/site/about/guidelines.xhtml

Statistical analysis

The case-crossover design was used to investigate the association between short-term exposure to air pollution and the occurrence of SIDS events controlling for average daily temperature and national holidays. This design, introduced by Maclure,[20] has been widely applied in air pollution studies and is particularly useful for estimating the risk of a rare acute outcome associated with short-term exposure.[24-27] In case-crossover design each case acts as their own control and like case-control studies[28] the distribution of exposure is compared between 'cases' and 'controls'. That is, exposure at the time just prior to the event ('case' or 'index' time) is compared with a set of 'control' times that represent the expected distribution of exposure for non-event follow-up times. The design helps primarily to control for confounding by subject-specific factors which remain static over time such as ethnicity and gender.

We applied the time stratified case-crossover approach where the strata are matching days based on the same day of the week, calendar month and year that has previously been used to minimise bias. [29] That is, control days were selected from the same day of the week, within the same calendar month and year as the event day. We applied a conditional Poisson regression model which has been shown to give equivalent estimates as the conditional logistic model but with the advantage of readily allowing for overdispersion and autocorrelation.[30] All our models assume a linear effect for air pollution as reported in previous studies[31 -33] while temperature effects are likely to be non-linear and were modelled using natural splines with 3 degrees of freedom.[34-36]

For peer review only - http://bmjopen.bmj.com/site/about/guidelines.xhtml

Sensitivity analyses

The primary aim was to investigate the risk of SIDS events in relation to air pollution on the previous day in single pollutant models. To examine sensitivities to our *a priori* model specification, additional lag structures were explored including single lags of 0,1,2,…,6 days and also the corresponding average of lags 0-1, 0-2, 0-3,…, 0-6. Moreover, the association between SIDS and air pollution was examined after adjusting for the effect of each of the pollutants PM_{10}, SO_2, NO_2, NO, CO and O_3 as a second pollutant in turn. The aim of this two-pollutant modelling approach was to account for potential confounding effect of co-pollutants. Further sensitivity analyses were performed by controlling for minimum temperature instead of the average temperature. We excluded NOx from the sensitivity analyses as it showed very strong correlation particularly with NO (r=0.96).

Results are presented as percentage increases in mortality with 95% confidence intervals (CIs) for an interquartile range (IQR) increase in pollutant concentration. Hypothesis tests were two-sided with a significance level of 0.05. All analyses were performed using the R statistical software; details of the packages used and sample line of code for fitting specific conditional Poisson regression model is given in the supplementary material.[37]

RESULTS

Descriptive statistics

Over the study period (1996–2006), there were 211 SIDS events across the four postcode areas (B, CV, WS and WV) included in the analyses which accounted for about approximately

For peer review only - http://bmjopen.bmj.com/site/about/guidelines.xhtml

5% of the days within the study period (Table 2). In the same period and location, a total of 943,937 live (single) births were registered.

For peer review only

For peer review only - http://bmjopen.bmj.com/site/about/guidelines.xhtml

Table 2: Average air pollution (µgm^{-3}), temperature (^{o}C) and IMD scores for selected West Midlands postcode areas with SIDS and birth counts, 1996-2006

Post town (area)	PM_{10}	SO_2	CO	O_3	NO_2	NO	NOx	Temp. (Min-Max)	IMD score	SIDS event (%)	Birth count (%)
Birmingham (B)	23.8	6.8	0.4	38.5	33.9	17.5	60	7.2-12.8	36.6	118 (55.9)	753844 (79.9)
Coventry (CV)	18.3	11.3	0.3	32.4	22.5	9.2	36.2	7.7-13.5	23.7	43 (20.4)	91393 (9.7)
Walsall (WS)					41.5		89.4	6.4-14.0	25.2	24 (11.4)	53532 (5.7)
Wolverhampton (WV)	23.8	9.3	0.5	38.9	29.5	18.9	59.5	8.4-12.3	33.4	26 (12.3)	45168 (4.8)
Total										211 (100)	943937 (100)

For peer review only

For peer review only - http://bmjopen.bmj.com/site/about/guidelines.xhtml

The daily average air pollution concentrations and their standard deviations are presented in Table 3 and show that average concentrations tended to be below UK air quality limits as defined by the EU Ambient Air Quality Directive.[38]

For peer review only - http://bmjopen.bmj.com/site/about/guidelines.xhtml

Table 3: Descriptive statistics for daily SIDS mortality counts, air pollution (µgm^{-3})*,* *temperature* (^{o}C) *and birth counts for West Midlands, 1996-2006*

Variable	Mean	SD	Median	IQR	Min	Max
SIDS count	<1	<1	0	0-0	0	2
Average temperature	10.2	5.4	10.2	6.2-14.5	-4.2	25.0
Max temperature	13.1	6.0	12.9	8.5-17.6	-2.1	29.6
Min temperature	7.4	5.1	7.5	3.6-11.4	-7.5	20.7
PM_{10}	23.4	11.6	20.2	15.7-28.2	4.0	128.9
SO_2	8.5	6.9	7.2	4.4-10.2	0.0	70.8
CO	0.4	0.2	0.4	0.3-0.5	0.1	3.5
O_3	38.1	18.2	38.0	25.4-50.4	1.3	115.8
NO_2	33.2	14.3	31.2	22.3-41.8	5.9	113.0
NO	16.9	23.8	9.0	5.4-17.9	0.4	314.9
NOx	64.5	49.3	51.1	35.2-76.4	7.9	569.2
Birth count[a]	290	34	296	266 - 314	183	387
IMD score	32.3	12.6	31.7	21.3-40.5	7.2	54.9

[a] Counts are for single births only

For peer review only

Table 4 shows that there was generally a strong correlation between the levels of pollutants investigated with the exception of the weaker correlation between O_3 and PM_{10} (r=-0.26) and with SO_2 (r=-0.34). What was also notable was the negative correlation between Ozone and the other pollutants and how in contrast to the other pollutants its positive correlation with temperature.

For peer review only - http://bmjopen.bmj.com/site/about/guidelines.xhtml

Table 4: Pairwise Pearson correlations coefficients (r) for pollutants and temperature

	PM_{10}	SO_2	CO	O_3	NO_2	NO	NOx
SO_2	0.53						
CO	0.56	0.59					
O_3	-0.26	-0.34	-0.56				
NO_2	0.66	0.56	0.76	-0.62			
NO	0.54	0.48	0.87	-0.58	0.73		
NOx	0.59	0.56	0.90	-0.62	0.86	0.96	
Temperature	-0.08	-0.24	-0.39	0.40	-0.40	-0.38	-0.40

For peer review only

Comparing the four postcode areas, Birmingham had the highest SIDS mortality (about 56%) and births rates (about 80%). Air pollution concentrations were more or less similar for Birmingham and Wolverhampton except slightly lower levels of NO_2 and higher levels of SO_2 observed for the latter. Coventry had the lowest pollution level with respect to all pollutants except SO_2. Walsall had measurements for NO_2 and NOx only which were very large compared to the other postcode areas (Table 2). Similarly, Birmingham and Wolverhampton had higher average deprivation scores compared to Coventry and Walsall (Table 2). Also, SIDS counts and pollution levels tended to be larger in highly deprived areas and near motorways and A-road networks which may be a reflection of population density (Figure 1; see Supplementary File: Figure S1). There was also indication of seasonal pattern for SIDS occurrence; the highest proportions of SIDS were observed in January and February (about 10%) and among the relatively colder months and in July (about 14%) among the warmer months (data not shown).

Case-crossover analysis

Figure 2 shows the estimated odds ratio (OR) (95% CI) for the association of SIDS events with each of the pollutants considered based on the conditional Poisson model. For an interquartile range increase in previous day pollutant concentration, the percentage increase (95% confidence interval) for the risk of SIDS death was 16 (6, 27); $p = 0.002$ for PM_{10}, 1 (-7, 10); $p = 0.83$ for SO_2, 5 (-4, 14); $p = 0.33$ for CO, -17 (-27, -6) ; $p = 0.004$ for O_3, 16 (2, 31); $p = 0.02$ for NO_2, 2 (-3, 8); $p = 0.47$ for NO and 8 (-1, 18); $p = 0.07$ for NOx after controlling for average temperature and national holidays. Therefore, considering pollutant levels at lag 1, significant association with increased risk of SIDS was indicated only for PM_{10} and NO_2 while a protective effect was apparent for O_3.

For peer review only - http://bmjopen.bmj.com/site/about/guidelines.xhtml

Sensitivity analyses

Lag structures: Analyses exploring relationships between SIDS and air pollution for other lag structures (lags 0, 2, 3,…, 6 and corresponding average of lags 0-1, 0-2, 0-3,…, 0-6) indicated associations were somewhat sensitive to the choice of lag (Figure 3). In the single lag models, there was an impression of stronger delayed effects for CO, NO_2, O_3, PM_{10} and SO_2 compared to relatively recent lags (lag 0 and lag 1). The effect of NO appeared to remain comparatively flat across lags. Relatively more consistent association across lags was observed for NO_2 and PM_{10} and to some extent for CO. Likewise, in average lag models, ORs tended to increase with averaging over more lags with exception of NO where such an effect was not observed. Results were also similar (at least qualitatively) after adjusting for minimum temperature instead of the average temperature (see Supplementary File: Figure S2).

Multi-pollutant models: Further investigation using two-pollutant models also showed associations were sensitive to control of other pollutants. In general, adjusting for co-pollutants appeared to attenuate ORs towards the null except for NO_2 and PM_{10} where effects remained to persist (Figure 4). Interestingly, after controlling for PM_{10}, the protective effect of O_3 and the adverse effect of NO_2 observed in the single pollutant models were not apparent for most of the lag choices. Similarly, none of the other pollutants showed marked association with SIDS after controlling for PM_{10} effects. The estimates from the multi-pollutant model should, however, be interpreted with caution as most of the pollutants were correlated.

For peer review only - http://bmjopen.bmj.com/site/about/guidelines.xhtml

COMMENTS

Summary of findings

In this study we hypothesised a delayed effect (lag 1) of air pollution on SIDS incidence and investigated additional lags in sensitivity analyses and in single and multi-pollutant models. Though confidence intervals were wide, we found evidence suggesting association of SIDS mortality with PM_{10} and NO_2 exposure. Compared to other pollutants, their effects persisted after controlling for co-pollutants and across the various lag structures investigated. The exception was controlling for O_3 did attenuate the risk estimates observed for both PM_{10} and NO_2. There were no consistent associations observed with exposure to the remaining pollutants investigated (CO, SO_2, NO, NOx). An exception was the protective effect observed in relation to O_3 exposure.

view only

Strengths and limitations

Previous studies have tended to focus on relatively longer-term exposure to air pollutants.[39-41] Our study is one of the first to use case-crossover methodology to explore the impact of air pollution on SIDS based on data from UK. Using an approach that is suitable for estimating the risk of a rare acute outcome such as SIDS events, we were able to investigate various lags and multi-pollutant models indicating delayed effects of pollutants that persisted after controlling for co-pollutants. However, measurement error for air pollution exposure a potential limitation in this study as we did not use personal measurements. Other epidemiological studies using data from ambient monitoring stations have shown a good level of correlation between daily personal exposure and daily ambient air pollution measurements,[36, 37] though

For peer review only - http://bmjopen.bmj.com/site/about/guidelines.xhtml

there is the potential for measurement error that can attenuate regression coefficient estimates. That is, such misclassification will likely lead to an under estimation of the effects. We were also unable to control for influenza and humidity as we did not have access to reliable data for these variables.

Detailed discussion

Certain groups of the population are more vulnerable to ambient air pollution than others and children figure predominantly amongst them due to the fragility of their immune system and the ratio of their lung capacity to their size. [42] Particulate matter has been recognised as the most significant contributor to air quality related morbidity and mortality [43] and a number of studies have reported a link between infant mortality and PM_{10} at similar concentrations of pollutant below air quality guidelines as are described in this study.[44-46, 11] However previous evidence of a direct association with SIDS is inconclusive and few studies describe a significant association of PM_{10} with increased risk. [6, 47]

The potential association with NO_2 we found reflected the findings of a multi-city study from Canada that found an increased risk for SIDS with a lag of two-days.[8] The issue of lag is interesting we found the strongest association with SIDS with PM_{10} and NO_2 occurred over several lags in line with previous studies that have found evidence of a lag between exposure to criteria pollutants and mortality. For PM_{10}, a single days exposure has been shown to have an effect for up to 5 days [48, 49] and numbers of respiratory deaths appear to be more affected by air pollution levels on previous days, than cardiovascular deaths that are impacted by same-day pollution.[50] For example myocardial infarction represents an acute response to a trigger.[51]

For peer review only - http://bmjopen.bmj.com/site/about/guidelines.xhtml

This is biologically plausible when considering how a wider and more lagged response can be expected for deaths from respiratory responses to pollution (eg, COPD) via the proposed mechanisms, such as pulmonary or systemic inflammation and modulated immunity.[52] With regards the apparent protective properties of O_3 that we describe here it's worth noting that these have been seen previously.[40] One possible reason for the negative association of O_3 and SIDS might be its negative correlation with other pollutants, in particular that with fine particulate matter pollution.[53] O_3 also showed a positive correlation with temperature while all other pollutants were natively correlated.

Concern around the effects of air pollution continue to mount particularly within developing economies [54] where it contributes to 3.3 million premature deaths worldwide per year a figure estimated to double by 2050 if the issue remains unattended.[55] However it appears that recently fossil fuel emissions have begun to increase again due to the developing world's reliance on them to power their expanding economies.[56] In the developed West, policy initiatives are beginning to recognise the mounting issue posed by the adverse effects on health posed by ambient air pollution. However shifting political priorities in the United States has seen a commitment to revitalise the coal industry [57] and an increase in the production of shale oil [58] However mitigating these risks is not a straightforward proposition and government policies appear slow to react, for example in the UK pledges to cease sales of diesel and petrol cars do not come into effect until 2040.[59]

In the absence of coherent policy to address the issue a number of proposals have been put forward to mitigate the effects of high levels of ambient exposure. Knowing that children and young adults may be highly susceptible to some of the subclinical changes caused by air

For peer review only - http://bmjopen.bmj.com/site/about/guidelines.xhtml

pollution [60,61] and as indoor concentrations are lower than ambient levels advice has been to remain indoors to reduce exposure and acute health risks on high air pollution days.[62] There are also systems available for cleaning indoor air though these may be deemed expensive for the economically deprived.[63] There has also been a case made for chemo-preventive interventions, such as antioxidant or antithrombotic agents, but without data on health outcomes, no recommendations can be made in their use for primary prevention. [64]

Conclusions

Understanding the effects of air pollution on child heath is more relevant than ever. Our work here has highlighted a potential association of Sudden Infant Death with PM_{10} and NO_2 and the association with particulate matter and infant mortality in particular is widely recognised. However, until policy reflects the growing evidence and responds to mounting public concern, it would appear to be the responsibility of individuals to take independent action to mitigate the effects of air pollution and protect the health of their young ones.

For peer review only

ACKNOWLEDGMENTS

Funding: This work was supported by The Lullaby Trust, grant number 260.

Competing interests: All authors declare: grants from The Lullaby Trust; no financial relationships with any organisations that might have an interest in the submitted work in the previous three years and no other relationships or activities that could appear to have influenced the submitted work.

Author contributions statement: IJL contributed to the conceptualisation of the study, initial draft manuscript and supervised the research project at all stages. NIM contributed to the research design, performed the data management, analysis and interpretation, and with IJL drafted the initial manuscript; JGA contributed to the conceptualisation of the study, data interpretation and critically reviewed the manuscript; JJKJ contributed to the conceptualisation of the study, data interpretation and critically reviewed the manuscript; All authors approved the final manuscript as submitted.

Transparency: As lead author, I can confirm that the manuscript is an honest, accurate, and transparent account of the study being reported. No important aspects have been omitted and there are no discrepancies from the planned study.

Ethics committee approval: Ethical approval was given by the University of Birmingham, Life and Health Sciences Ethical Review Committee.

Data sharing: No additional data available.

For peer review only

REFERENCES

1. Royal College of Physicians, Royal College of Paediatrics and Child Health. Every breath we take: The lifelong impact of air pollution. 2016. https://www.rcplondon.ac.uk/projects/outputs/every-breath-we-take-lifelong-impact-air-pollution (accessed Apr 2017).

2. World Health Organization. Burden of disease from Ambient Air Pollution for 2012. 2014. http://www.who.int/phe/health_topics/outdoorair/databases/AAP_BoD_results_March2014.pdf?ua=1 (accessed Apr 2017).

3. Brauer M, Lencar C, Tamburic L, et al. A cohort study of traffic-related air pollution impacts on birth outcomes. Environ Health Perspect 2008;116(5):680-6.

4. Fleischer NL, Merialdi M, van Donkelaar A, et al. Outdoor air pollution, preterm birth, and low birth weight: analysis of the World Health Organization global survey on maternal and perinatal health. Environ Health Perspect 2014;122(4):425-30.

5. Knobel HH, Chen CJ, Liang KY. Sudden infant death syndrome in relation to weather and optimetrically measured air pollution in Taiwan. Pediatrics 1995;96(6):1106-10.

6. Lipfert FW, Zhang J, Wyzga RE. Infant mortality and air pollution: a comprehensive analysis of U.S. data for 1990. J Air Waste Manag Assoc 2000;50(8):1350-66.

7. Hunt CE. Sudden infant death syndrome and other causes of infant mortality: diagnosis, mechanisms, and risk for recurrence in siblings. Am J Respir Crit Care Med 2001;164(3):346-57.

8. Dales R, Burnett RT, Smith-Doiron M, et al. Air pollution and sudden infant death syndrome. Pediatrics 2004;113(6):e628-31.

For peer review only - http://bmjopen.bmj.com/site/about/guidelines.xhtml

9. Kaiser R, Romieu I, Medina S, et al. Air pollution attributable postneonatal infant mortality in U.S. metropolitan areas: a risk assessment study. Environ Health 2004;3(1):4.

10. Heinrich J, Slama R. Fine particles, a major threat to children. Int J Hyg Environ Health 2007;210(5):617-22.

11. Yorifuji T, Kashima S, Doi H. Acute exposure to fine and coarse particulate matter and infant mortality in Tokyo, Japan (2002-2013). Sci Total Environ. 2016 May 1;551-552:66-72. doi:.1016/j.scitotenv.2016.01.211. Epub 2016 Feb 11.

12. Luechinger S Air pollution and infant mortality: A natural experiment from power plant desulfurization Journal of Health Economics 37 (2014) 219–231

13. National Institute of Child Health and Human Development. How many infants die from SIDS or are at risk for SIDS? 2013. https://www.nichd.nih.gov/health/topics/sids/conditioninfo/pages/risk.aspx (accessed Apr 2017).

14. Kinney HC, Thach BT. The sudden infant death syndrome. N Engl J Med 2009;361(8):795-805.

15. Goldwater PN Infection: the neglected paradigm in SIDS research Archives of Disease in Childhood 2017;102:767-772.

16. Leach CE, Blair PS, Fleming PJ, et al. Epidemiology of SIDS and explained sudden infant deaths. CESDI SUDI Research Group. Pediatrics 1999;104(4):e43.

17. Brooke H, Gibson A, Tappin D, et al. Case-control study of sudden infant death syndrome in Scotland, 1992-5. BMJ 1997;314(7093):1516-20.

18. Douglas AS, Allan TM, Helms PJ. Seasonality and the Sudden Infant Death Syndrome during 1987-9 and 1991-3 in Australia and Britain. BMJ 1996;312(7043):1381-3.

For peer review only

For peer review only - http://bmjopen.bmj.com/site/about/guidelines.xhtml

19. Glinianaia SV, Rankin J, Bell R, et al. Does particulate air pollution contribute to infant death? A systematic review. Environ Health Perspect 2004;112(14):1365-70.

20. Tong S, Colditz P. Air pollution and sudden infant death syndrome: a literature review. Paediatr Perinat Epidemiol 2004;18(5):327-35.

21. "2014 UK Population Estimates". ONS. Retrieved 27 July 2015

22. National Centre for Atmospheric Science, Natural Environment Research Council. British Atmospheric Data Centre. http://badc.nerc.ac.uk/home/index_beacon.html (accessed Apr 2017).

23. Pope A. "Indices of Deprivation England and Wales 2010" [ESRI Shapefile geospatial data]. 2013. https://www.sharegeo.ac.uk/handle/10672/481 (accessed Dec 2016).

24. Maclure M. The case-crossover design: a method for studying transient effects on the risk of acute events. Am J Epidemiol 1991;133(2):144-53.

25. Lee JT, Schwartz J. Reanalysis of the effects of air pollution on daily mortality in Seoul, Korea: A case-crossover design. Environ Health Perspect 1999;107(8):633-6.

26. Jaakkola JJ. Case-crossover design in air pollution epidemiology. Eur Respir J Suppl 2003;40:81s-5s.

27. Pope CA 3rd, Muhlestein JB, May HT, et al. Ischemic heart disease events triggered by short-term exposure to fine particulate air pollution. Circulation 2006;114(23):2443-8.

28. Bhaskaran K, Hajat S, Armstrong B, et al. The effects of hourly differences in air pollution on the risk of myocardial infarction: case crossover analysis of the MINAP database. BMJ 2011;343:d5531.

29. Janes H, Sheppard L, Lumley T. Case-crossover analyses of air pollution exposure data: referent selection strategies and their implications for bias. Epidemiology 2005;16:717-26

For peer review only

For peer review only - http://bmjopen.bmj.com/site/about/guidelines.xhtml

30. Breslow NE, Day NE. Statistical methods in cancer research. Volume I - The analysis of case-control studies. IARC Sci Publ 1980;32:5-338.

31. Armstrong BG, Gasparrini A, Tobias A. Conditional Poisson models: a flexible alternative to conditional logistic case cross-over analysis. BMC Med Res Methodol 2014;14:122.

32. Le Tertre A, Medina S, Samoli E, et al. Short-term effects of particulate air pollution on cardiovascular diseases in eight European cities. J Epidemiol Community Health 2002;56(10):773-9.

33. Katsouyanni K, Samet JM, Anderson HR, et al. Air pollution and health: a European and North American approach (APHENA). Res Rep Health Eff Inst 2009;142:5-90.

34. Scheers H, Mwalili SM, Faes C, et al. Does air pollution trigger infant mortality in Western Europe? A case-crossover study. Environ Health Perspect 2011;119(7):1017-22.

35. Braga AL, Zanobetti A, Schwartz J. The effect of weather on respiratory and cardiovascular deaths in 12 U.S. cities. Environ Health Perspect 2002;110(9):859-63.

36. Armstrong B. Models for the relationship between ambient temperature and daily mortality. Epidemiology 2006;17(6):624-31.

37. R Core Team. R: A Language and Environment for Statistical Computing. 2013. http://www.R-project.org (accessed Apr 2017).

38. https://uk-air.defra.gov.uk/air-pollution/uk-eu-limits (accessed Dec 2017).

39. R Core Team. R: A Language and Environment for Statistical Computing. 2013. http://www.R-project.org (accessed Apr 2017).

40. Ritz B, Wilhelm M, Zhao Y. Air pollution and infant death in southern California, 1989-2000. Pediatrics 2006;118(2):493-502.

For peer review only - http://bmjopen.bmj.com/site/about/guidelines.xhtml

41. Woodruff TJ, Darrow LA, Parker JD. Air pollution and postneonatal infant mortality in the United States, 1999-2002. Environ Health Perspect 2008;116(1):110-5.

42. Pope CA, Dockery DW Health Effects of Fine Particulate Air Pollution: Lines that Connect Journal of the Air & Waste Management Association 2006; 56 (6)

43. Lancet commission on air quality and health Published online October 19, 2017 http://dx.doi.org/10.1016/S0140-6736(17)32345-0 (Accessed November 2017)

44. Rice MB, Ljungman PL, Wilker EH, et al. Short-term exposure to air pollution and lung function in the Framingham Heart Study. Am J Respir Crit Care Med 2013; 188 (11): 1351- 357.

45. Samoli E, Stafoggia M, Rodopoulou S, et al. Associations between fine and coarse particles and mortality in Mediterranean cities: results from the MED-PARTICLES project. Environ Health Perspect 2013; 121 (8):932–938

46. Scheers H, Mwalili SM, Faes C, Fierens F, Nemery B, and Nawrot TS. Does Air Pollution Trigger Infant Mortality in Western Europe? A Case-Crossover Study Environ Health Perspect 2011; 119:1017–1022

47. Woodruff TJ, Parker JD, Schoendorf KC. Fine Particulate Matter (PM(2.5)) Air Pollution and Selected Causes of Postneonatal Infant Mortality in California. Environ Health Perspect 2006;114(5):786-90.

48. Schwartz J The distributed lag between air pollution and daily deaths Epidemiology. 2000 May;11(3):320-6

49. Association Between Short-Term Exposure to PM2.5 and PM10 and Mortality in Susceptible Subgroups: A Multisite Case-Crossover Analysis of Individual Effect Modifiers Rita E Massimo A, Annunziata S, Giovanna F, Canova BC, De Togni A, Di

For peer review only

Biagio K, Gherardi B, Giannini S, Lauriola P American Journal of Epidemiology, Volume 184, Issue 10, 15 November 2016, Pages 744–754

50. Braga AL, Zanobetti A, Schwartz J. The lag structure between particulate air pollution and respiratory and cardiovascular deaths in 10 US cities. J Occup Environ Med. 2001 Nov;43(11):927-33

51. Gold DR, Litonjua A, Schwartz J, et al. Ambient pollution and heart rate variability. Circulation. 2000; 101: 1267–1273

52. Blackwell C, Moscovis S, Hall S, et al. Exploring the risk factors for sudden infant deaths and their role in inflammatory responses to infection. Front Immunol. 2015;6:44.

53. Sarnat J, Schwartz J, Suh H. Comment on: Fine particulate air pollution and mortality in 20 U.S. cities. N Engl J Med 2001;344(16):1253-4

54. WHO (2014) "Burden of Disease from Ambient Air Pollution for 2012." n.d. (http://www.who.int/phe/health_topics/outd oorair/databases/AAP_BoD_results_March2014.pdf)

55. Lelieveld, J., J. S. Evans, M. Fnais, D. Giannadaki, and A. Pozzer. 2015. "The Contribution of Outdoor Air Pollution Sources to Premature Mortality on a Global Scale." Nature 525 (7569). nature.com: 367–71

56. Jackson R, Le Quere C, Andrew RM, Canadell JG, Peters GP, Roy J, Wu L Environ. Res. Lett. 12 (2017) 110202

57. http://www.independent.co.uk/news/world/americas/donald-trump-coal-mining-jobs-promise-experts-disagree-executive-order-a7656486.html (accessed November 2017)

58. https://www.eia.gov/tools/faqs/faq.php?id=847&t=6 (accessed November 2017)

For peer review only - http://bmjopen.bmj.com/site/about/guidelines.xhtml

59. https://www.theguardian.com/politics/2017/jul/25/britain-to-ban-sale-of-all-diesel-and-petrol-cars-and-vans-from-2040 (accessed November 2017)

60. Rich DQ, Kipen HM, Huang W, et al. Association between changes in air pollution levels during the Beijing Olympics and biomarkers of inflammation and thrombosis in healthy young adults. JAMA 2012;307:2068-78

61. Wright RJ, Brunst KJ. Programming of respiratory health in childhood: influence of outdoor air pollution. Curr Opin Pediatr 2013;25:232-9.

62. Plaia A, Ruggieri M: Air quality indices: a review. Rev Environ Sci Bio 2011;10:165-79

63. David L. MacIntosh , Theodore A. Myatt , Jerry F. Ludwig , Brian J. Baker , Helen H. Suh & John D. Spengler (2008) Whole House Particle Removal and Clean Air Delivery Rates for In-Duct and Portable Ventilation Systems, Journal of the Air & Waste Management Association, 58:11, 1474-1482

64. Laumbach R, Meng Q, Kipen H. What can individuals do to reduce personal health risks from air pollution? J Thorac Dis 2015;7(1):96-107. doi: 10.3978/ j.issn.2072-1439.2014.12.2

FIGURE LEGENDS

Fig. 1 Air pollution monitoring sites with road networks (motorways and A-roads) and SIDS events in the study area. We used ArcGIS Desktop 10.2 (http://www.esri.com/software/arcgis) to create the map combining shapefiles for postcode areas and motorways and overlaying air pollution and SIDS data.

For peer review only - http://bmjopen.bmj.com/site/about/guidelines.xhtml

Fig. 2 Estimated risk of SIDS for an IQR increase in lag 1 pollutant concentration before and after controlling for confounding by average temperature and national holidays

Fig. 3 Risk of SIDS associated with an IQR increase in pollution for selected lags and average of lags; models adjusted for average temperature and national holidays

Fig. 4 Risk of SIDS associated with an IQR increase in air pollution after controlling for co-pollutants, average temperature and national holidays(panel titles indicate: main and co-pollutant respectively)

For peer review only

For peer review only

For peer review only - http://bmjopen.bmj.com/site/about/guidelines.xhtml

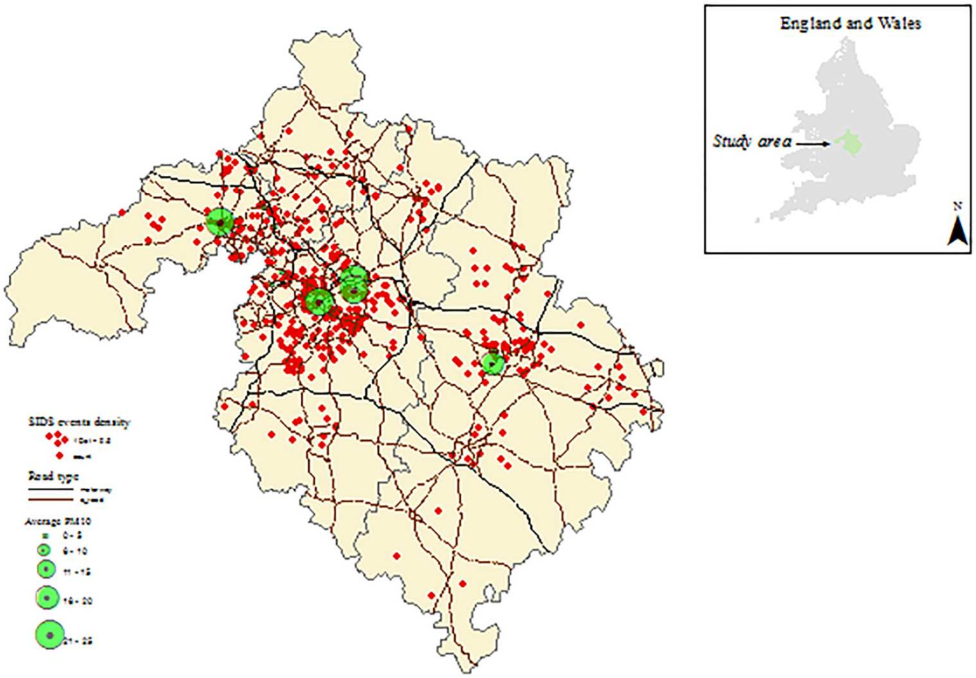

Fig. 1 Air pollution monitoring sites with road networks (motorways and A-roads) and SIDS events in the study area. We used ArcGIS Desktop 10.2 (http://www.esri.com/software/arcgis) to create the map combining shapefiles for postcode areas and motorways and overlaying air pollution and SIDS data.

152x105mm (300 x 300 DPI)

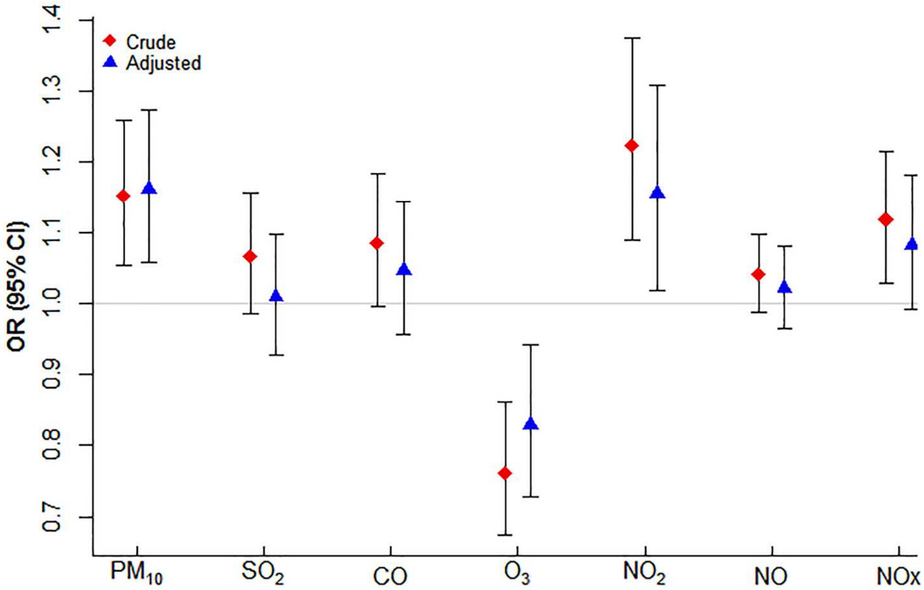

Fig. 2 Estimated risk of SIDS for an IQR increase in lag 1 pollutant concentration before and after controlling for confounding by average temperature and national holidays

179x143mm (300 x 300 DPI)

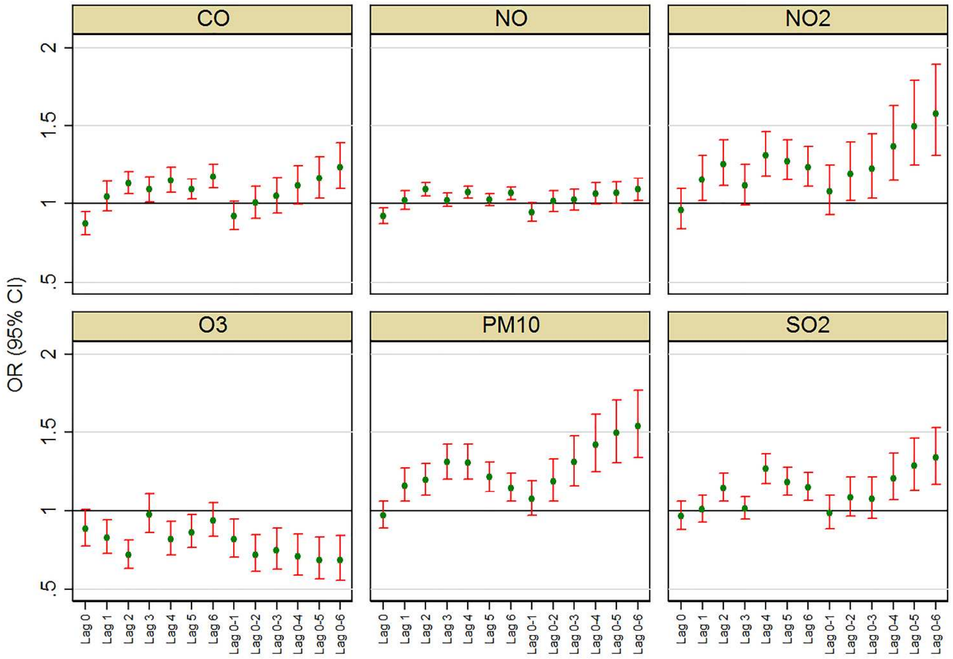

Fig. 3 Risk of SIDS associated with an IQR increase in pollution for selected lags and average of lags; models adjusted for average temperature and national holidays

475x346mm (300 x 300 DPI)

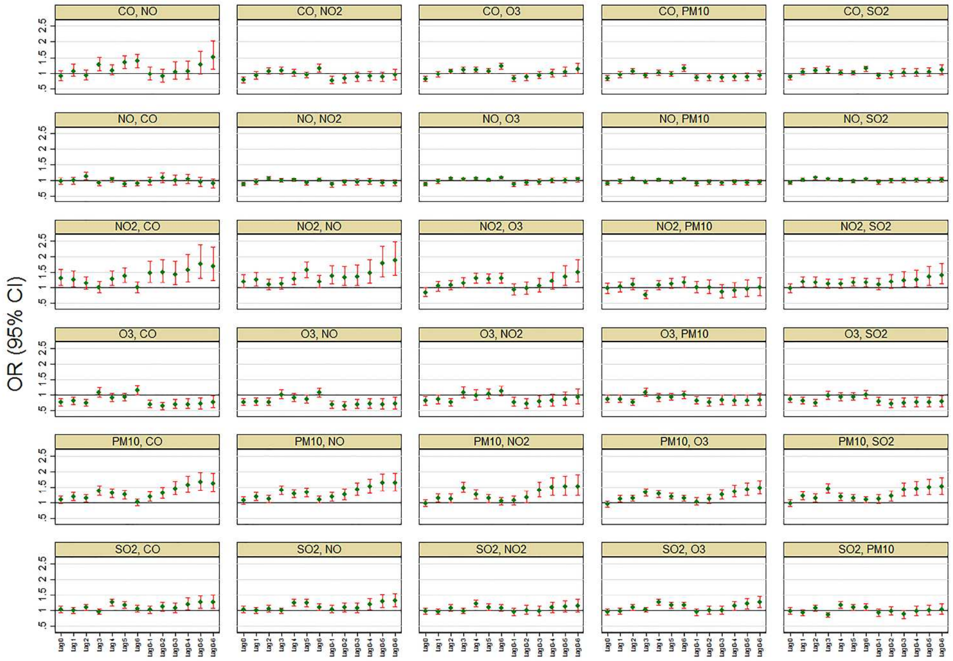

Fig. 4 Risk of SIDS associated with an IQR increase in air pollution after controlling for co-pollutants, average temperature and national holidays(panel titles indicate: main and co-pollutant respectively)

475x346mm (300 x 300 DPI)

For peer review only - http://bmjopen.bmj.com/site/about/guidelines.xhtml

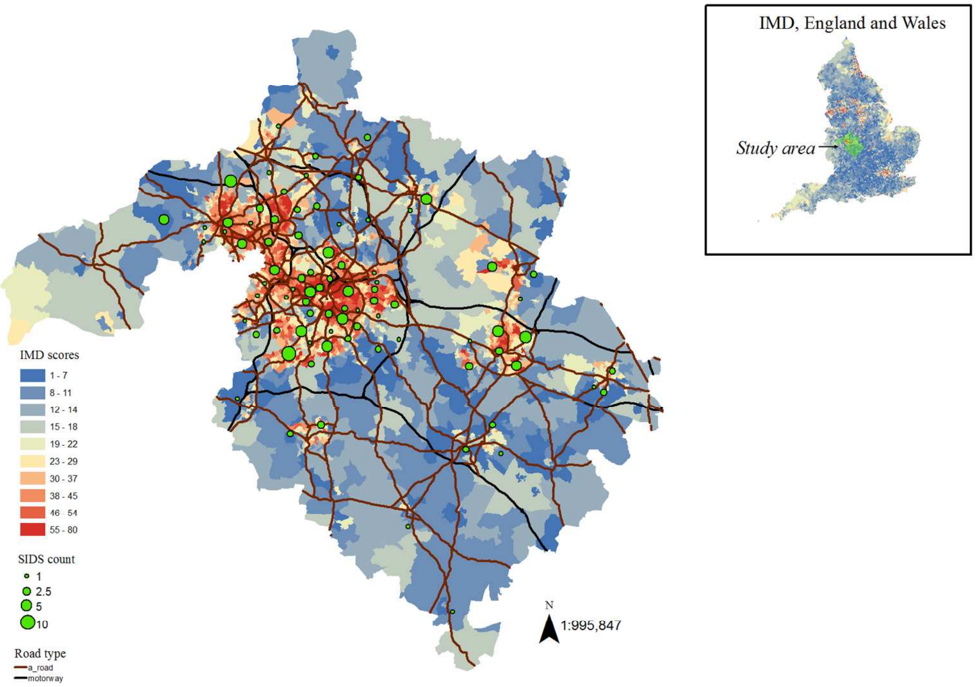

152x114mm (300 x 300 DPI)

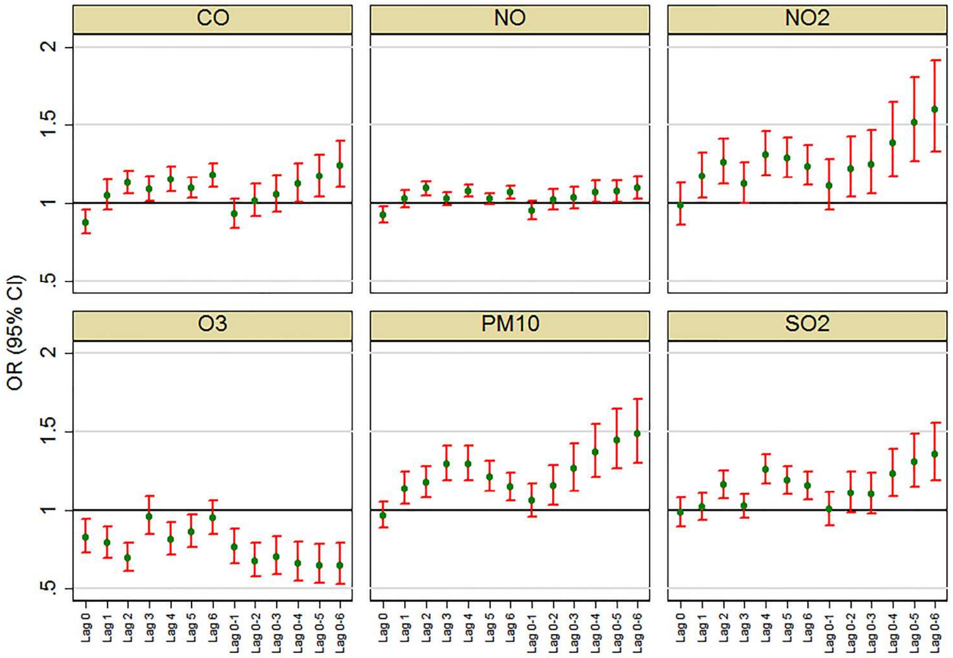

327x238mm (300 x 300 DPI)

STROBE (STrengthening the Reporting of OBservational studies in Epidemiology) Checklist for authors of CORR

This table is modified from and used with the permission of The STROBE Initiative, www.strobe-statement.org.

	Item No	Recommendation	Please insert check where included or N/A where not applicable
**Title and abstract**	1	(*a*) Indicate the study's design with a commonly used term in the title or the abstract	√
		(*b*) Provide in the abstract an informative and balanced summary of what was done and what was found	√
**Introduction**			
Background/rationale	2	Explain the scientific background and rationale for the investigation being reported	Page 4
Objectives	3	State specific objectives, including any prespecified hypotheses	Page 8
**Methods**			
Study design	4	Present key elements of study design early in the paper	Page 8/9
Setting	5	Describe the setting, locations, and relevant dates, including periods of recruitment, treatment, follow-up, and data collection	Tbc
Participants	6	(*a*) *Cohort study*—Give the eligibility criteria, and the sources and methods of selection of participants. Describe methods of follow-up<br>*Case-control study*—Give the eligibility criteria, and the sources and methods of case ascertainment and control selection. Give the rationale for the choice of cases and controls<br>*Cross-sectional study*—Give the eligibility criteria, and the sources and methods of selection of participants	Page 6
		(*b*) *Cohort study*—For matched studies, give matching criteria and number of treated and untreated<br>*Case-control study*—For matched studies, give matching criteria and the number of controls per case	Page 9

For peer review only

Participants	13*	(a) Report numbers of individuals at each stage of study—eg numbers potentially eligible, examined for eligibility, confirmed eligible, included in the study, completing follow-up, and analysed	(page 11 –descriptive statistics)
		(b) Give reasons for non-participation at each stage	
Descriptive data	14*	(a) Give characteristics of study participants (eg demographic, clinical, social) and information on other treatments and potential confounders	(Table 2)
		(b) Indicate number of participants with missing data for each variable of interest	n/a
		(c) *Cohort study*—Summarise follow-up time (eg, average and total amount)	
Variables	7	Clearly define all outcomes, treatments, predictors, potential confounders, and effect modifiers. Give diagnostic criteria, if applicable	Table 2 and 3
Data sources/ measurement	8	For each variable of interest, give sources of data and details of methods of assessment (measurement). Describe comparability of assessment methods if there is more than one group	Page 6 and Page 8
Bias	9	Describe any efforts to address potential sources of bias	Page 9
Study size	10	Explain how the study size was arrived at	Page 6 – Data on SIDS mortality
Quantitative variables	11	Explain how quantitative variables were handled in the analyses. If applicable, describe which groupings were chosen and why	Page 9
Statistical methods	12	(*a*) Describe all statistical methods, including those used to control for confounding	Page 8/9
		(*b*) Describe any methods used to examine subgroups and interactions	n/a
		(*c*) Explain how missing data were addressed	n/a
		(*d*) *Cohort study*—If applicable, explain how loss to follow-up was addressed<br>*Case-control study*—If applicable, explain how matching of cases and controls was addressed<br>*Cross-sectional study*—If applicable, describe analytical methods taking account of sampling strategy	Page 8/9
		(*e*) Describe any sensitivity analyses	

For peer review only

Is Ambient Air Pollution associated with onset of Sudden Infant Death Syndrome: A case crossover study in the UK

Journal:	*BMJ Open*
Manuscript ID	bmjopen-2017-018341.R2
Article Type:	Research
Date Submitted by the Author:	02-Feb-2018
Complete List of Authors:	Litchfield, Ian; University of Birmingham, Institute of Applied Health Research<br>Ayres, Jon; University of Birmingham, Institute of Applied Health Research<br>Jaakkola, Jouni; University of Oulu, Center for Environmental and Respiratory Health Research<br>Mohammed, Nuredin; University of Birmingham, Institute of Applied Health Research
**Primary Subject Heading**:	Epidemiology
Secondary Subject Heading:	Occupational and environmental medicine, Paediatrics, Public health, Respiratory medicine
Keywords:	EPIDEMIOLOGY, Community child health < PAEDIATRICS, Cot death < PAEDIATRICS, Paediatric thoracic medicine < PAEDIATRICS, PUBLIC HEALTH

SCHOLARONE™
Manuscripts

For peer review only - http://bmjopen.bmj.com/site/about/guidelines.xhtml

Is Ambient Air Pollution associated with onset of Sudden Infant Death Syndrome: A case crossover study in the UK

Ian J Litchfield[1*], Jon G Ayres[1], Jouni J K Jaakkola[2], and Nuredin I Mohammed[1,3]

Affiliations

1 Institute of Applied Health Research, College of Medical and Dental Sciences
University of Birmingham
Edgbaston, Birmingham, B15 2TT
United Kingdom

2 Center for Environmental and Respiratory Health Research
Faculty of Medicine
POB 5000 (Aapistie 5B)
90014 Oulu, Finland

3 Medical Research Council Unit The Gambia
Atlantic Road
PO Box 273, Banjul
The Gambia

***Corresponding Author**

Dr Ian J Litchfield

Address:	Institute of Applied Health Research, College of Medical and Dental Sciences<br>University of Birmingham<br>Edgbaston, Birmingham, B15 2TT<br>United Kingdom
Email:	i.litchfield@bham.ac.uk
Telephone:	+44 0121 414 6006
Fax:	+44 0121 414 6217

For peer review only

Word count: 3024

Number of figures: 4

Number of tables: 4

For peer review only

For peer review only - http://bmjopen.bmj.com/site/about/guidelines.xhtml

ABSTRACT

Objectives: Air pollution has been associated with increased mortality and morbidity in several studies with indications its effect could be more severe in children. This study examined the relationship between short-term variations in criteria air pollutants and occurrence of sudden infant death syndrome (SIDS).

Design: We used a case-crossover study design which is widely applied in air pollution studies and particularly useful for estimating the risk of a rare acute outcome associated with short-term exposure.

Setting: The study used data from the West Midlands region in the UK.

Participants: We obtained daily time series data on SIDS mortality (ICD-9: 798.0 or ICD-10: R95) for the period 1996-2006 with a total of 211 SIDS events.

Primary outcome measures: Daily counts of SIDS events.

Results: For an interquartile range increase in previous day pollutant concentration, the percentage increases (95% confidence interval) in SIDS were 16 (6, 27) for PM_{10}, 1 (-7, 10) for SO_2, 5 (-4, 14) for CO, -17 (-27, -6) for O_3, 16 (2, 31) for NO_2, and 2 (-3, 8) for NO after controlling for average temperature and national holidays. PM_{10} and NO_2 showed relatively consistent association which persisted across different lag structures and after adjusting for co-pollutants.

Conclusions: The results indicated ambient air pollutants, particularly PM_{10} and NO_2, may show an association with increased SIDS mortality. Thus, future studies are recommended to understand possible mechanistic explanations on the role of air pollution on SIDS incidence and the ways in which we might reduce pollution exposure among infants.

Clinical trial registration: Not applicable.

Keywords: Air pollution; Child health; Time-series; Infant mortality.

Article Summary

Strengths and limitations of this study

- Sudden infant death is the leading cause of death in healthy infants between one month and one year olds and our study is the first based on time series data from the UK to investigate the relationship between common air pollutants and SIDS.
- Though our study is limited in power due to the comparatively small number of daily SIDS events our chosen design is widely applied in air pollution studies and particularly useful for estimating the risk of a rare acute outcome associated with short-term exposure.

INTRODUCTION

The quality of ambient air is an important factor in the health of adults and children. Ambient air quality is the second largest challenge facing public health in the UK.[1] According

to the World Health Organisation (WHO), over 3.7 million premature deaths per annum may be attributed to the harmful effects of ambient air. Children it would seem are more vulnerable than any other group[2] and recent studies have indicated how even low-levels of traffic-related air pollution can have a negative impact on birth outcomes and perinatal health.[3, 4] A number of studies have also suggested a link between ambient air quality and the incidence of sudden infant death syndrome (SIDS).[5-12] Here we examine the effects of the short term variations in air pollution and the onset of SIDS.

Sudden infant death is the leading cause of death in healthy infants between one month and one year old,[13] and the impact on the families is notably traumatic as the death is without warning or witness.[14] The exact cause continues to tax researchers [15] though is likely the result of a combination of factors including susceptibility and environmental stressors such as lower social status of parents,[16] environmental tobacco smoke,[16, 17] the prone position[18] and the winter season.[5, 18] The impact of tobacco smoke suggests a respiratory trigger may be involved, however the evidence of an association between SIDS and air pollution warrant further research as findings from epidemiological studies have been inconsistent.[19, 20] and few have satisfactorily explored the impact of short-term exposure on SIDS. Our study is the first based on time series data from the UK to investigate the relationship between common air pollutants and SIDS. We have collated data from a ten-year period on concentrations of air pollution and onset of SIDS within the West Midlands one of the largest and most polluted conurbations in the UK and have conducted a case-crossover study to determine any associations.

For peer review only

METHODS

Settings

The West Midlands is a metropolitan county in the centre of the UK. It has a population of some 2.8 million. [21] The West Midlands is one of the most heavily urbanized counties in the UK and forms the most populated conurbation in the UK outside London, It is at the heart of the UK motorway network and remains a significant centre of the UK's manufacturing industry.

Data collection

We combined data on SIDS events with data on total births, air pollution, air temperature and a measure of deprivation.

Data on SIDS mortality and total births

We obtained daily time series data on SIDS mortality (ICD-9: 798.0 or ICD-10: R95) for the period 1996-2006 from the Perinatal Institute (PI). All cases were between 0 and 12 months old at onset. This data consisted of the date of death and the first three digits of the postcode to avoid the possibility of identifying individual cases due to the rare nature of SIDS. This allowed us to explore the effects of short-term exposure. We obtained daily births data with West Midlands' postcodes from the Office of National Statistics (ONS) for the period 1996-2006 and was used for descriptive analysis only.

Air pollution data

The daily time series data on air pollution were compiled from the UK air quality archive managed by Department for the Environment, Food and Rural Affairs (DEFRA). These include a total of ten monitoring stations in the West Midlands measuring PM_{10}, SO_2, NO_2, NO, NOx, CO and O_3 including sites within Birmingham, Coventry, Walsall and Wolverhampton. Before 1996 the reliability of air quality data was inconsistent. Not all monitoring stations had measurements for the range of pollutants over the entire study period and the precise pollutants measured at each monitoring centre are described in Table 1.

Table 1: Pollutants measured and corresponding time period by monitoring stations

Name (postcode area)	Pollutants	Time period[a]
Birmingham Centre (B)	PM_{10}, SO_2, NO_2,NO, NOx, CO, O_3	01/01/1996-31/12/2006
Birmingham East (B)	PM_{10}, SO_2, NO_2,NO, CO, O_3	01/01/1996-03/08/2004
Birmingham Tyburn (B)	PM_{10}, SO_2, NO_2,NO, CO, O_3	16/08/2004-31/12/2006
Oldbury (B)	PM_{10}, SO_2, NO_2,NO, NOx, CO, O_3	27/06/1997-20/09/1998
West Bromwich (B)	PM_{10}, SO_2, NO_2,NO, NOx, CO, O_3	04/11/1998-31/12/2006
Coventry Centre (CV)	PM_{10}, SO_2, NO_2,NO, NOx, CO, O_3	19/02/1997-31/12/2006
Coventry Memorial (CV)	PM_{10}, SO_2, NO_2,NO, NOx, CO, O_3	26/02/2001-31/12/2006
Walsall Alumwell (WS)	NO_2, NOx	01/01/1996-31/12/2006
Walsall Willenhall (WS)	NO_2	13/05/1997-31/12/2006
Wolverhampton Centre (WV)	PM_{10}, SO_2, NO_2,NO, NOx, CO, O_3	19/12/1995-31/12/2006

[a]Period with at least one pollutant being measured and taking into account missing data between start and end dates.

For peer review only - http://bmjopen.bmj.com/site/about/guidelines.xhtml

We aimed to examine the association between day-to-day variability in air pollution exposure and SIDS counts over the entire region rather than contrasting exposure and outcome between areas within the West Midlands region. Therefore the daily pollution data for West Midlands were calculated by averaging across all monitoring stations with available measurements as our models were based on the temporal relationship between air pollution and SIDS; we did not fit a spatial model.

Temperature data

Data on daily minimum and maximum temperature were compiled for weather monitoring stations in the West Midlands from the Meteorological Office British Atmospheric Data Centre (BADC).[22] We used the average daily temperature which was calculated by taking the average of the minimum and maximum temperature at each monitoring station for each day to obtain the daily average temperature for the entire West Midlands.

Index of multiple deprivation (IMD) score

The IMD score is a composite measure based on seven dimensions of deprivation including income deprivation, employment deprivation, health deprivation and disability, education deprivation, crime deprivation, barriers to housing and services deprivation and living environment deprivation. The data for the 2010 IMD at postcode level were downloaded from EDINA Digimap ShareGeo facility, an online spatial data repository and was used for descriptive analysis only.[23]

For peer review only - http://bmjopen.bmj.com/site/about/guidelines.xhtml

Statistical analysis

The case-crossover design was used to investigate the association between short-term exposure to air pollution and the occurrence of SIDS events controlling for average daily temperature and national holidays. This design, introduced by Maclure,[20] has been widely applied in air pollution studies and is particularly useful for estimating the risk of a rare acute outcome associated with short-term exposure.[24-27] In case-crossover design each case acts as their own control and like case-control studies[28] the distribution of exposure is compared between 'cases' and 'controls'. That is, exposure at the time just prior to the event ('case' or 'index' time) is compared with a set of 'control' times that represent the expected distribution of exposure for non-event follow-up times. The design helps primarily to control for confounding by subject-specific factors which remain static over time such as ethnicity and gender.

We applied the time stratified case-crossover approach which has previously been used to minimise bias. [29] That is, control days were selected from the same day of the week, within the same calendar month and year as the event day. We applied a conditional Poisson regression model which has been shown to give equivalent estimates as the conditional logistic model but with the advantage of readily allowing for overdispersion and autocorrelation.[30] All our models assume a linear effect for air pollution as reported in previous studies[31 -33] while temperature effects are likely to be non-linear and were modelled using natural splines with 3 degrees of freedom.[34-36]

Sensitivity analyses

For peer review only - http://bmjopen.bmj.com/site/about/guidelines.xhtml

The primary aim was to investigate the risk of SIDS events in relation to air pollution on the previous day in single pollutant models. To examine sensitivities to our *a priori* model specification, additional lag structures were explored including single lags of 0,1,2,…,6 days and also the corresponding average of lags 0-1, 0-2, 0-3,…, 0-6. Moreover, the association between SIDS and air pollution was examined after adjusting for the effect of each of the pollutants PM_{10}, SO_2, NO_2, NO, CO and O_3 as a second pollutant in turn. The aim of this two-pollutant modelling approach was to account for potential confounding effect of co-pollutants. Further sensitivity analyses were performed by controlling for minimum temperature instead of the average temperature. We excluded NOx from the sensitivity analyses as it showed very strong correlation particularly with NO (r=0.96).

Results are presented as percentage increases in mortality with 95% confidence intervals (CIs) for an interquartile range (IQR) increase in pollutant concentration. Hypothesis tests were two-sided with a significance level of 0.05. All analyses were performed using the R statistical software; details of the packages used and sample line of code for fitting specific conditional Poisson regression model is given in the supplementary material (See Supplementary File:S1).[37]

RESULTS

Descriptive statistics

Over the study period (1996–2006), there were 211 SIDS events across the four postcode areas (B, CV, WS and WV) included in the analyses which accounted for about approximately

For peer review only - http://bmjopen.bmj.com/site/about/guidelines.xhtml

5% of the days within the study period (Table 2). In the same period and location, a total of 943,937 live (single) births were registered.

For peer review only

Table 2: Average air pollution (µgm^{-3}), temperature (^{o}C) and IMD scores for selected West Midlands postcode areas with SIDS and birth counts, 1996-2006

Post town (area)	PM_{10}	SO_2	CO	O_3	NO_2	NO	NOx	Temp. (Min-Max)	IMD score	SIDS event (%)	Birth count (%)
Birmingham (B)	23.8	6.8	0.4	38.5	33.9	17.5	60	7.2-12.8	36.6	118 (55.9)	753844 (79.9)
Coventry (CV)	18.3	11.3	0.3	32.4	22.5	9.2	36.2	7.7-13.5	23.7	43 (20.4)	91393 (9.7)
Walsall (WS)					41.5		89.4	6.4-14.0	25.2	24 (11.4)	53532 (5.7)
Wolverhampton (WV)	23.8	9.3	0.5	38.9	29.5	18.9	59.5	8.4-12.3	33.4	26 (12.3)	45168 (4.8)
Total										211 (100)	943937 (100)

For peer review only

The daily average air pollution concentrations and their standard deviations are presented in Table 3 and show that average concentrations tended to be below UK air quality limits as defined by the EU Ambient Air Quality Directive.[38]

For peer review only - http://bmjopen.bmj.com/site/about/guidelines.xhtml

Table 3: Descriptive statistics for daily SIDS mortality counts, air pollution (μgm^{-3}), temperature (^{o}C) and birth counts for West Midlands, 1996-2006

Variable	Mean	SD	Median	IQR	Min	Max
SIDS count	<1	<1	0	0-0	0	2
Average temperature	10.2	5.4	10.2	6.2-14.5	-4.2	25.0
Max temperature	13.1	6.0	12.9	8.5-17.6	-2.1	29.6
Min temperature	7.4	5.1	7.5	3.6-11.4	-7.5	20.7
PM_{10}	23.4	11.6	20.2	15.7-28.2	4.0	128.9
SO_2	8.5	6.9	7.2	4.4-10.2	0.0	70.8
CO	0.4	0.2	0.4	0.3-0.5	0.1	3.5
O_3	38.1	18.2	38.0	25.4-50.4	1.3	115.8
NO_2	33.2	14.3	31.2	22.3-41.8	5.9	113.0
NO	16.9	23.8	9.0	5.4-17.9	0.4	314.9
NOx	64.5	49.3	51.1	35.2-76.4	7.9	569.2
Birth count[a]	290	34	296	266 - 314	183	387
IMD score	32.3	12.6	31.7	21.3-40.5	7.2	54.9

[a] Counts are for single births only

For peer review only

Table 4 shows that there was generally a strong correlation between the levels of pollutants investigated with the exception of the weaker correlation between O_3 and PM_{10} (r=-0.26) and with SO_2 (r=-0.34). What was also notable was the negative correlation between Ozone and the other pollutants and how in contrast to the other pollutants its positive correlation with temperature.

For peer review only - http://bmjopen.bmj.com/site/about/guidelines.xhtml

Table 4: Pairwise Pearson correlations coefficients (r) for pollutants and temperature

	PM_{10}	SO_2	CO	O_3	NO_2	NO	NOx
SO_2	0.53						
CO	0.56	0.59					
O_3	-0.26	-0.34	-0.56				
NO_2	0.66	0.56	0.76	-0.62			
NO	0.54	0.48	0.87	-0.58	0.73		
NOx	0.59	0.56	0.90	-0.62	0.86	0.96	
Temperature	-0.08	-0.24	-0.39	0.40	-0.40	-0.38	-0.40

For peer review only - http://bmjopen.bmj.com/site/about/guidelines.xhtml

Comparing the four postcode areas, Birmingham had the highest SIDS mortality (about 56%) and births rates (about 80%). Air pollution concentrations were more or less similar for Birmingham and Wolverhampton except slightly lower levels of NO_2 and higher levels of SO_2 observed for the latter. Coventry had the lowest pollution level with respect to all pollutants except SO_2. Walsall had measurements for NO_2 and NOx only which were very large compared to the other postcode areas (Table 2). Similarly, Birmingham and Wolverhampton had higher average deprivation scores compared to Coventry and Walsall (Table 2). Also, SIDS counts and pollution levels tended to be larger in highly deprived areas and near motorways and A-road networks which may be a reflection of population density (Figure 1; see Supplementary File: Figure S2). There was also indication of seasonal pattern for SIDS occurrence; the highest proportions of SIDS were observed in January and February (about 10%) and among the relatively colder months and in July (about 14%) among the warmer months (data not shown).

Case-crossover analysis

Figure 2 shows the estimated odds ratio (OR) (95% CI) for the association of SIDS events with each of the pollutants considered based on the conditional Poisson model. For an interquartile range increase in previous day pollutant concentration, the percentage increase (95% confidence interval) for the risk of SIDS death was 16 (6, 27); $p = 0.002$ for PM_{10}, 1 (-7, 10); $p = 0.83$ for SO_2, 5 (-4, 14); $p = 0.33$ for CO, -17 (-27, -6) ; $p = 0.004$ for O_3, 16 (2, 31); $p = 0.02$ for NO_2, 2 (-3, 8); $p = 0.47$ for NO and 8 (-1, 18); $p = 0.07$ for NOx after controlling for average temperature and national holidays. Therefore, considering pollutant levels at lag 1, significant association with increased risk of SIDS was indicated only for PM_{10} and NO_2 while a protective effect was apparent for O_3.

For peer review only - http://bmjopen.bmj.com/site/about/guidelines.xhtml

Sensitivity analyses

Lag structures: Analyses exploring relationships between SIDS and air pollution for other lag structures (lags 0, 2, 3,…, 6 and corresponding average of lags 0-1, 0-2, 0-3,…, 0-6) indicated associations were somewhat sensitive to the choice of lag (Figure 3). In the single lag models, there was an impression of stronger delayed effects for CO, NO_2, O_3, PM_{10} and SO_2 compared to relatively recent lags (lag 0 and lag 1). The effect of NO appeared to remain comparatively flat across lags. Relatively more consistent association across lags was observed for NO_2 and PM_{10} and to some extent for CO. Likewise, in average lag models, ORs tended to increase with averaging over more lags with exception of NO where such an effect was not observed. Results were also similar (at least qualitatively) after adjusting for minimum temperature instead of the average temperature (see Supplementary File: Figure S3).

Multi-pollutant models: Further investigation using two-pollutant models also showed associations were sensitive to control of other pollutants. In general, adjusting for co-pollutants appeared to attenuate ORs towards the null except for NO_2 and PM_{10} where effects remained to persist (Figure 4). Interestingly, after controlling for PM_{10}, the protective effect of O_3 and the adverse effect of NO_2 observed in the single pollutant models were not apparent for most of the lag choices. Similarly, none of the other pollutants showed marked association with SIDS after controlling for PM_{10} effects. The estimates from the multi-pollutant model should, however, be interpreted with caution as most of the pollutants were correlated.

COMMENTS

Summary of findings

In this study we hypothesised a delayed effect (lag 1) of air pollution on SIDS incidence and investigated additional lags in sensitivity analyses and in single and multi-pollutant models. Though confidence intervals were wide, we found evidence suggesting association of SIDS mortality with PM_{10} and NO_2 exposure. Compared to other pollutants, their effects persisted after controlling for co-pollutants and across the various lag structures investigated. The exception was controlling for O_3 did attenuate the risk estimates observed for both PM_{10} and NO_2. There were no consistent associations observed with exposure to the remaining pollutants investigated (CO, SO_2, NO, NOx). An exception was the protective effect observed in relation to O_3 exposure.

Strengths and limitations

Previous studies have tended to focus on relatively longer-term exposure to air pollutants.[37,39,40] Our study is one of the first to use case-crossover methodology to explore the impact of air pollution on SIDS based on data from UK. Using an approach that is suitable for estimating the risk of a rare acute outcome such as SIDS events, we were able to investigate various lags and multi-pollutant models indicating delayed effects of pollutants that persisted after controlling for co-pollutants. However, measurement error for air pollution exposure a potential limitation in this study as we did not use personal measurements. Other epidemiological studies using data from ambient monitoring stations have shown a good level of correlation between daily personal exposure and daily ambient air pollution measurements,[36, 37] though

view only

For peer review only - http://bmjopen.bmj.com/site/about/guidelines.xhtml

there is the potential for measurement error that can attenuate regression coefficient estimates. That is, such misclassification will likely lead to an under estimation of the effects. We were also unable to control for influenza and humidity as we did not have access to reliable data for these variables.

Detailed discussion

Certain groups of the population are more vulnerable to ambient air pollution than others and children figure predominantly amongst them due to the fragility of their immune system and the ratio of their lung capacity to their size. [41] Particulate matter has been recognised as the most significant contributor to air quality related morbidity and mortality [42] and a number of studies have reported a link between infant mortality and PM_{10} at similar concentrations of pollutant below air quality guidelines as are described in this study.[11,43,44] However previous evidence of a direct association with SIDS is inconclusive and few studies describe a significant association of PM_{10} with increased risk. [6, 45]

The potential association with NO_2 we discovered reflected the findings of a multi-city study from Canada that found an increased risk for SIDS with a lag of two-days.[8] More broadly our findings are in line with previous studies that have found evidence of a lag between exposure to criteria pollutants and mortality. For PM_{10}, a single days exposure has been shown to have an effect for up to 5 days [46, 47] and numbers of respiratory deaths appear to be more affected by air pollution levels on previous days, than cardiovascular deaths that are impacted by same-day pollution.[48] For example myocardial infarction represents an acute response to a trigger.[49]

For peer review only - http://bmjopen.bmj.com/site/about/guidelines.xhtml

This is biologically plausible when considering how a wider and more lagged response can be expected for deaths from respiratory responses to pollution (eg, COPD) via the proposed mechanisms, such as pulmonary or systemic inflammation and modulated immunity.[50] With regards the apparent protective properties of O_3 that we describe here it's worth noting that these have been seen previously.[39] One possible reason for the negative association of O_3 and SIDS might be its negative correlation with other pollutants, in particular that with fine particulate matter pollution.[51] O_3 also showed a positive correlation with temperature while all other pollutants were negatively correlated.

Concern around the effects of air pollution continue to mount particularly within developing economies [52] where it contributes to 3.3 million premature deaths worldwide per year a figure estimated to double by 2050 if the issue remains unattended.[53] However it appears that recently fossil fuel emissions have begun to increase again due to the developing world's reliance on them to power their expanding economies.[54] In the developed West, policy initiatives are beginning to recognise the mounting issue posed by the adverse effects on health posed by ambient air pollution. However shifting political priorities in the United States has seen a commitment to revitalise the coal industry [55] and an increase in the production of shale oil [56] However mitigating these risks is not a straightforward proposition and government policies appear slow to react, for example in the UK pledges to cease sales of diesel and petrol cars do not come into effect until 2040.[57]

In the absence of coherent policy to address the issue a number of proposals have been put forward to mitigate the effects of high levels of ambient exposure. Knowing that children and young adults may be highly susceptible to some of the subclinical changes caused by air

For peer review only - http://bmjopen.bmj.com/site/about/guidelines.xhtml

pollution [58,59] and as indoor concentrations are lower than ambient levels advice has been to remain indoors to reduce exposure and acute health risks on high air pollution days.[60] There are also systems available for cleaning indoor air though these may be deemed expensive for the economically deprived.[61] There has also been a case made for chemo-preventive interventions, such as antioxidant or antithrombotic agents, but without data on health outcomes, no recommendations can be made in their use for primary prevention. [62]

Conclusions

Understanding the effects of air pollution on child heath is more relevant than ever. Our work here has highlighted a potential association of Sudden Infant Death with PM_{10} and NO_2 and the association with particulate matter and infant mortality in particular is widely recognised. However, until policy reflects the growing evidence and responds to mounting public concern, it would appear to be the responsibility of individuals to take independent action to mitigate the effects of air pollution and protect the health of their young ones.

For peer review only

ACKNOWLEDGMENTS

Funding: This work was supported by The Lullaby Trust, grant number 260.

Competing interests: All authors declare: grants from The Lullaby Trust; no financial relationships with any organisations that might have an interest in the submitted work in the previous three years and no other relationships or activities that could appear to have influenced the submitted work.

Author contributions statement: IJL contributed to the conceptualisation of the study, initial draft manuscript and supervised the research project at all stages. NIM contributed to the research design, performed the data management, analysis and interpretation, and with IJL drafted the initial manuscript; JGA contributed to the conceptualisation of the study, data interpretation and critically reviewed the manuscript; JJKJ contributed to the conceptualisation of the study, data interpretation and critically reviewed the manuscript; All authors approved the final manuscript as submitted.

Transparency: As lead author, I can confirm that the manuscript is an honest, accurate, and transparent account of the study being reported. No important aspects have been omitted and there are no discrepancies from the planned study.

Ethics committee approval: Ethical approval was given by the University of Birmingham, Life and Health Sciences Ethical Review Committee.

Data sharing: No additional data available.

For peer review only

REFERENCES

1. Royal College of Physicians, Royal College of Paediatrics and Child Health. Every breath we take: The lifelong impact of air pollution. 2016. https://www.rcplondon.ac.uk/projects/outputs/every-breath-we-take-lifelong-impact-air-pollution (accessed Apr 2017).

2. World Health Organization. Burden of disease from Ambient Air Pollution for 2012. 2014. http://www.who.int/phe/health_topics/outdoorair/databases/AAP_BoD_results_March2014.pdf?ua=1 (accessed Apr 2017).

3. Brauer M, Lencar C, Tamburic L, et al. A cohort study of traffic-related air pollution impacts on birth outcomes. Environ Health Perspect 2008;116(5):680-6.

4. Fleischer NL, Merialdi M, van Donkelaar A, et al. Outdoor air pollution, preterm birth, and low birth weight: analysis of the World Health Organization global survey on maternal and perinatal health. Environ Health Perspect 2014;122(4):425-30.

5. Knobel HH, Chen CJ, Liang KY. Sudden infant death syndrome in relation to weather and optimetrically measured air pollution in Taiwan. Pediatrics 1995;96(6):1106-10.

6. Lipfert FW, Zhang J, Wyzga RE. Infant mortality and air pollution: a comprehensive analysis of U.S. data for 1990. J Air Waste Manag Assoc 2000;50(8):1350-66.

7. Hunt CE. Sudden infant death syndrome and other causes of infant mortality: diagnosis, mechanisms, and risk for recurrence in siblings. Am J Respir Crit Care Med 2001;164(3):346-57.

8. Dales R, Burnett RT, Smith-Doiron M, et al. Air pollution and sudden infant death syndrome. Pediatrics 2004;113(6):e628-31.

For peer review only

9. Kaiser R, Romieu I, Medina S, et al. Air pollution attributable postneonatal infant mortality in U.S. metropolitan areas: a risk assessment study. Environ Health 2004;3(1):4.

10. Heinrich J, Slama R. Fine particles, a major threat to children. Int J Hyg Environ Health 2007;210(5):617-22.

11. Yorifuji T, Kashima S, Doi H. Acute exposure to fine and coarse particulate matter and infant mortality in Tokyo, Japan (2002-2013). Sci Total Environ. 2016 May 1;551-552:66-72. doi:.1016/j.scitotenv.2016.01.211. Epub 2016 Feb 11.

12. Luechinger S Air pollution and infant mortality: A natural experiment from power plant desulfurization Journal of Health Economics 37 (2014) 219–231

13. National Institute of Child Health and Human Development. How many infants die from SIDS or are at risk for SIDS? 2013. https://www.nichd.nih.gov/health/topics/sids/conditioninfo/pages/risk.aspx (accessed Apr 2017).

14. Kinney HC, Thach BT. The sudden infant death syndrome. N Engl J Med 2009;361(8):795-805.

15. Goldwater PN Infection: the neglected paradigm in SIDS research Archives of Disease in Childhood 2017;102:767-772.

16. Leach CE, Blair PS, Fleming PJ, et al. Epidemiology of SIDS and explained sudden infant deaths. CESDI SUDI Research Group. Pediatrics 1999;104(4):e43.

17. Brooke H, Gibson A, Tappin D, et al. Case-control study of sudden infant death syndrome in Scotland, 1992-5. BMJ 1997;314(7093):1516-20.

18. Douglas AS, Allan TM, Helms PJ. Seasonality and the Sudden Infant Death Syndrome during 1987-9 and 1991-3 in Australia and Britain. BMJ 1996;312(7043):1381-3.

For peer review only

19. Glinianaia SV, Rankin J, Bell R, et al. Does particulate air pollution contribute to infant death? A systematic review. Environ Health Perspect 2004;112(14):1365-70.

20. Tong S, Colditz P. Air pollution and sudden infant death syndrome: a literature review. Paediatr Perinat Epidemiol 2004;18(5):327-35.

21. "2014 UK Population Estimates". ONS. Retrieved 27 July 2015

22. National Centre for Atmospheric Science, Natural Environment Research Council. British Atmospheric Data Centre. http://badc.nerc.ac.uk/home/index_beacon.html (accessed Apr 2017).

23. Pope A. "Indices of Deprivation England and Wales 2010" [ESRI Shapefile geospatial data]. 2013. https://www.sharegeo.ac.uk/handle/10672/481 (accessed Dec 2016).

24. Maclure M. The case-crossover design: a method for studying transient effects on the risk of acute events. Am J Epidemiol 1991;133(2):144-53.

25. Lee JT, Schwartz J. Reanalysis of the effects of air pollution on daily mortality in Seoul, Korea: A case-crossover design. Environ Health Perspect 1999;107(8):633-6.

26. Jaakkola JJ. Case-crossover design in air pollution epidemiology. Eur Respir J Suppl 2003;40:81s-5s.

27. Pope CA 3rd, Muhlestein JB, May HT, et al. Ischemic heart disease events triggered by short-term exposure to fine particulate air pollution. Circulation 2006;114(23):2443-8.

28. Bhaskaran K, Hajat S, Armstrong B, et al. The effects of hourly differences in air pollution on the risk of myocardial infarction: case crossover analysis of the MINAP database. BMJ 2011;343:d5531.

29. Janes H, Sheppard L, Lumley T. Case-crossover analyses of air pollution exposure data: referent selection strategies and their implications for bias. Epidemiology 2005;16:717-26

For peer review only

30. Breslow NE, Day NE. Statistical methods in cancer research. Volume I - The analysis of case-control studies. IARC Sci Publ 1980;32:5-338.

31. Armstrong BG, Gasparrini A, Tobias A. Conditional Poisson models: a flexible alternative to conditional logistic case cross-over analysis. BMC Med Res Methodol 2014;14:122.

32. Le Tertre A, Medina S, Samoli E, et al. Short-term effects of particulate air pollution on cardiovascular diseases in eight European cities. J Epidemiol Community Health 2002;56(10):773-9.

33. Katsouyanni K, Samet JM, Anderson HR, et al. Air pollution and health: a European and North American approach (APHENA). Res Rep Health Eff Inst 2009;142:5-90.

34. Scheers H, Mwalili SM, Faes C, et al. Does air pollution trigger infant mortality in Western Europe? A case-crossover study. Environ Health Perspect 2011;119(7):1017-22.

35. Braga AL, Zanobetti A, Schwartz J. The effect of weather on respiratory and cardiovascular deaths in 12 U.S. cities. Environ Health Perspect 2002;110(9):859-63.

36. Armstrong B. Models for the relationship between ambient temperature and daily mortality. Epidemiology 2006;17(6):624-31.

37. R Core Team. R: A Language and Environment for Statistical Computing. 2013. http://www.R-project.org (accessed Apr 2017).

38. https://uk-air.defra.gov.uk/air-pollution/uk-eu-limits (accessed Dec 2017).

39. Ritz B, Wilhelm M, Zhao Y. Air pollution and infant death in southern California, 1989-2000. Pediatrics 2006;118(2):493-502.

40. Woodruff TJ, Darrow LA, Parker JD. Air pollution and postneonatal infant mortality in the United States, 1999-2002. Environ Health Perspect 2008;116(1):110-5.

41. Pope CA, Dockery DW Health Effects of Fine Particulate Air Pollution: Lines that Connect Journal of the Air & Waste Management Association 2006; 56 (6)

For peer review only

For peer review only - http://bmjopen.bmj.com/site/about/guidelines.xhtml

42. Lancet commission on air quality and health Published online October 19, 2017 http://dx.doi.org/10.1016/S0140-6736(17)32345-0 (Accessed November 2017)

43. Rice MB, Ljungman PL, Wilker EH, et al. Short-term exposure to air pollution and lung function in the Framingham Heart Study. Am J Respir Crit Care Med 2013; 188 (11): 1351- 357.

44. Samoli E, Stafoggia M, Rodopoulou S, et al. Associations between fine and coarse particles and mortality in Mediterranean cities: results from the MED-PARTICLES project. Environ Health Perspect 2013; 121 (8):932–938

45. Woodruff TJ, Parker JD, Schoendorf KC. Fine Particulate Matter (PM(2.5)) Air Pollution and Selected Causes of Postneonatal Infant Mortality in California. Environ Health Perspect 2006;114(5):786-90.

46. Schwartz J The distributed lag between air pollution and daily deaths Epidemiology. 2000 May;11(3):320-6

47. Association Between Short-Term Exposure to PM2.5 and PM10 and Mortality in Susceptible Subgroups: A Multisite Case-Crossover Analysis of Individual Effect Modifiers Rita E Massimo A, Annunziata S, Giovanna F, Canova BC, De Togni A, Di Biagio K, Gherardi B, Giannini S, Lauriola P American Journal of Epidemiology, Volume 184, Issue 10, 15 November 2016, Pages 744–754

48. Braga AL, Zanobetti A, Schwartz J. The lag structure between particulate air pollution and respiratory and cardiovascular deaths in 10 US cities. J Occup Environ Med. 2001 Nov;43(11):927-33

49. Gold DR, Litonjua A, Schwartz J, et al. Ambient pollution and heart rate variability. Circulation. 2000; 101: 1267–1273

For peer review only - http://bmjopen.bmj.com/site/about/guidelines.xhtml

50. Blackwell C, Moscovis S, Hall S, et al. Exploring the risk factors for sudden infant deaths and their role in inflammatory responses to infection. Front Immunol. 2015;6:44.

51. Sarnat J, Schwartz J, Suh H. Comment on: Fine particulate air pollution and mortality in 20 U.S. cities. N Engl J Med 2001;344(16):1253-4

52. WHO (2014) "Burden of Disease from Ambient Air Pollution for 2012." n.d. (http://www.who.int/phe/health_topics/outd oorair/databases/AAP_BoD_results_March2014.pdf)

53. Lelieveld, J., J. S. Evans, M. Fnais, D. Giannadaki, and A. Pozzer. 2015. "The Contribution of Outdoor Air Pollution Sources to Premature Mortality on a Global Scale." Nature 525 (7569). nature.com: 367–71

54. Jackson R, Le Quere C, Andrew RM, Canadell JG, Peters GP, Roy J, Wu L Environ. Res. Lett. 12 (2017) 110202

55. http://www.independent.co.uk/news/world/americas/donald-trump-coal-mining-jobs-promise-experts-disagree-executive-order-a7656486.html (accessed November 2017)

56. https://www.eia.gov/tools/faqs/faq.php?id=847&t=6 (accessed November 2017)

57. https://www.theguardian.com/politics/2017/jul/25/britain-to-ban-sale-of-all-diesel-and-petrol-cars-and-vans-from-2040 (accessed November 2017)

58. Rich DQ, Kipen HM, Huang W, et al. Association between changes in air pollution levels during the Beijing Olympics and biomarkers of inflammation and thrombosis in healthy young adults. JAMA 2012;307:2068-78

59. Wright RJ, Brunst KJ. Programming of respiratory health in childhood: influence of outdoor air pollution. Curr Opin Pediatr 2013;25:232-9.

60. Plaia A, Ruggieri M: Air quality indices: a review. Rev Environ Sci Bio 2011;10:165-79

For peer review only

61. David L. MacIntosh , Theodore A. Myatt , Jerry F. Ludwig , Brian J. Baker , Helen H. Suh & John D. Spengler (2008) Whole House Particle Removal and Clean Air Delivery Rates for In-Duct and Portable Ventilation Systems, Journal of the Air & Waste Management Association, 58:11, 1474-1482

62. Laumbach R, Meng Q, Kipen H. What can individuals do to reduce personal health risks from air pollution? J Thorac Dis 2015;7(1):96-107. doi: 10.3978/ j.issn.2072-1439.2014.12.2

FIGURE LEGENDS

Fig. 1 Air pollution monitoring sites with road networks (motorways and A-roads) and SIDS events in the study area. We used ArcGIS Desktop 10.2 (http://www.esri.com/software/arcgis) to create the map combining shapefiles for postcode areas and motorways and overlaying air pollution and SIDS data.

Fig. 2 Estimated risk of SIDS for an IQR increase in lag 1 pollutant concentration before and after controlling for confounding by average temperature and national holidays

Fig. 3 Risk of SIDS associated with an IQR increase in pollution for selected lags and average of lags; models adjusted for average temperature and national holidays

Fig. 4 Risk of SIDS associated with an IQR increase in air pollution after controlling for co-pollutants, average temperature and national holidays(panel titles indicate: main and co-pollutant respectively)

For peer review only - http://bmjopen.bmj.com/site/about/guidelines.xhtml

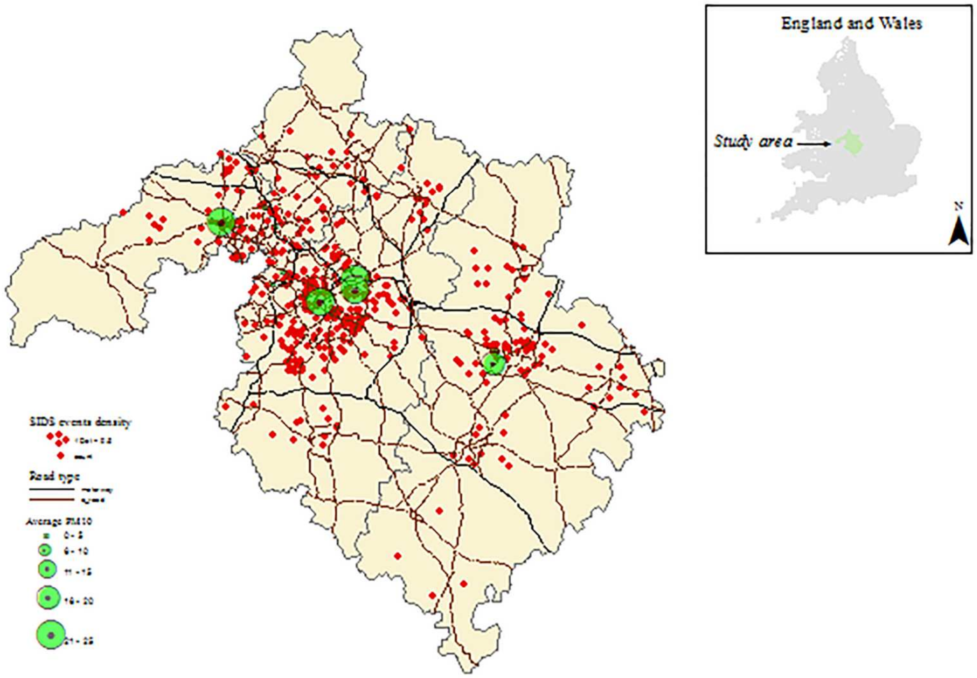

Fig. 1 Air pollution monitoring sites with road networks (motorways and A-roads) and SIDS events in the study area. We used ArcGIS Desktop 10.2 (http://www.esri.com/software/arcgis) to create the map combining shapefiles for postcode areas and motorways and overlaying air pollution and SIDS data.

152x105mm (300 x 300 DPI)

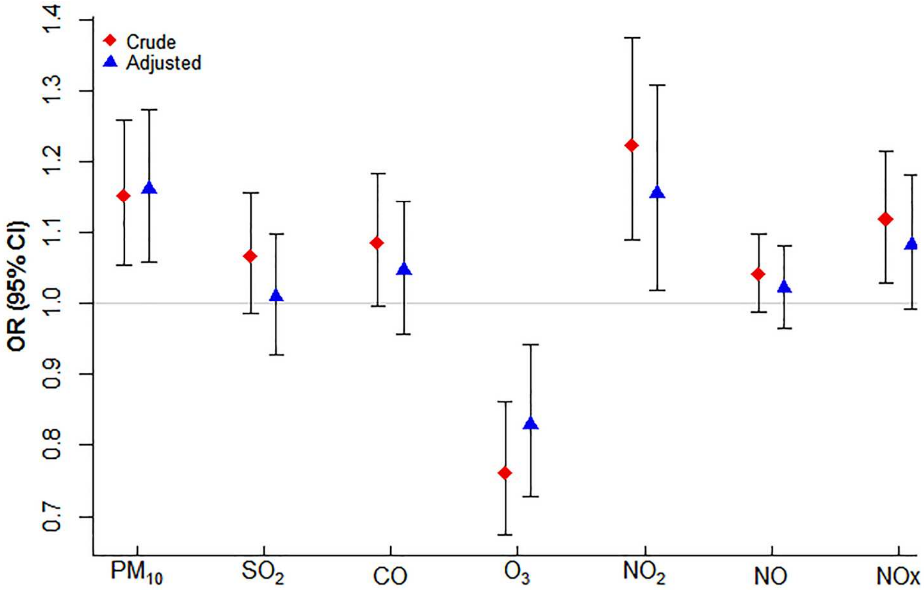

Fig. 2 Estimated risk of SIDS for an IQR increase in lag 1 pollutant concentration before and after controlling for confounding by average temperature and national holidays

179x143mm (300 x 300 DPI)

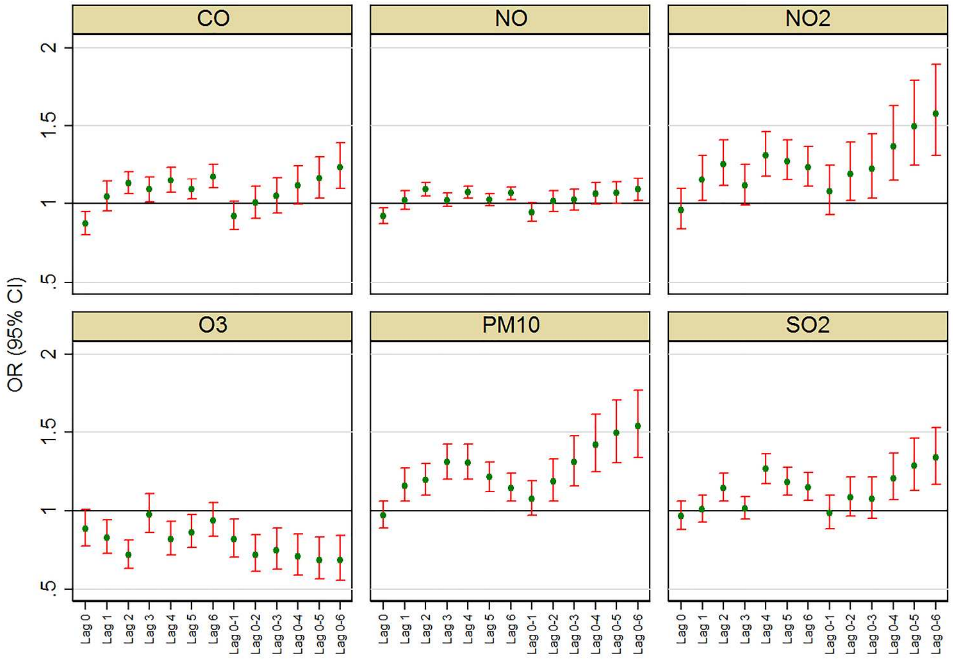

Fig. 3 Risk of SIDS associated with an IQR increase in pollution for selected lags and average of lags; models adjusted for average temperature and national holidays

475x346mm (300 x 300 DPI)

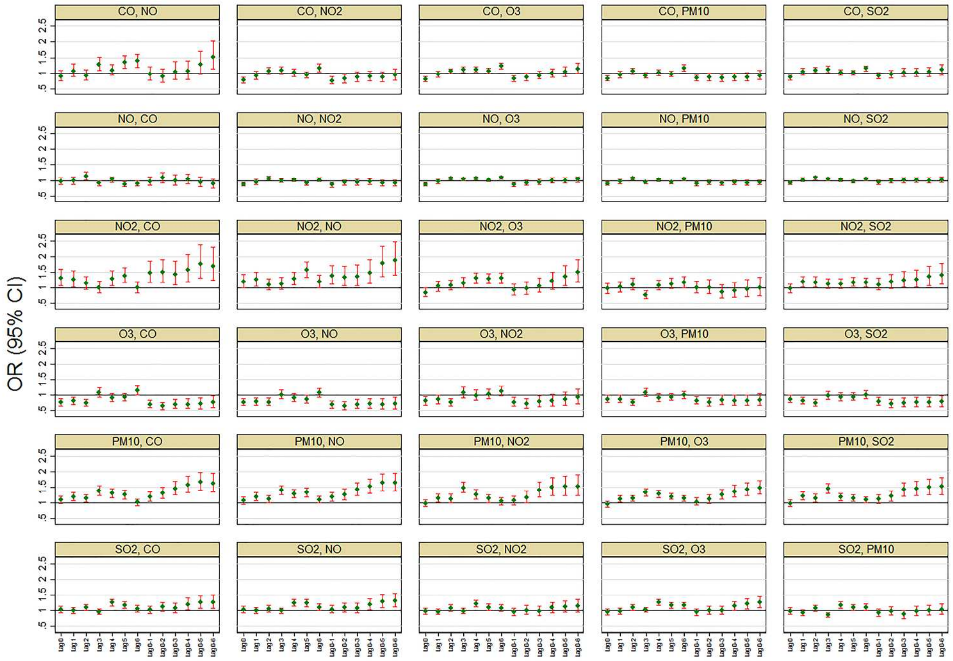

Fig. 4 Risk of SIDS associated with an IQR increase in air pollution after controlling for co-pollutants, average temperature and national holidays(panel titles indicate: main and co-pollutant respectively)

475x346mm (300 x 300 DPI)

Conditional Poisson Regression (CPR)

Here we applied the time stratified case-crossover approach where the strata are matching days based on the same day of the week, calendar month and year that has previously been used to minimise bias [Janes et al. 2005]. That is, control days were selected from the same day of the week, within the same calendar month and year as the event day. We applied a conditional Poisson regression model which has been shown to give equivalent estimates as the conditional logistic model but with the advantage of readily allowing for overdispersion. Thus, to fit the CPR model we first create the starta for yearXmonthXday-of-week from the original time series data and then fit the regression using the 'gnm' package adjusting for temprture spline and holday effects, conditional on starta as detailed in Armstrong et al. [2014]. An example line of R code to fit the CPR model using the R package 'gnm' is given below: *CPR_model <- gnm(sids_freq ~ Lag(pollution, k) + ns(temperature,3) + holiday, data=data, family=quasipoisson, eliminate=factor(stratum))*

where

Lag(pollution, k): pollution exposure at lag k

ns(temperature,3): temperature splines

sids_freq: SIDS count

stratum: stratum indicator (for yearXmonthXday-of-week)

References

Holly Janes, Lianne Sheppard, Thomas Lumley. Case–Crossover Analyses of Air Pollution Exposure Data, Referent Selection Strategies and Their Implications for Bias. Epidemiology 2005;16: 717-726.

Armstrong BG, Gasparrini A, Tobias A. Conditional Poisson models: a flexible alternative to conditional logistic case cross-over analysis. BMC Med Res Methodol 2014;14:122.

For peer review only

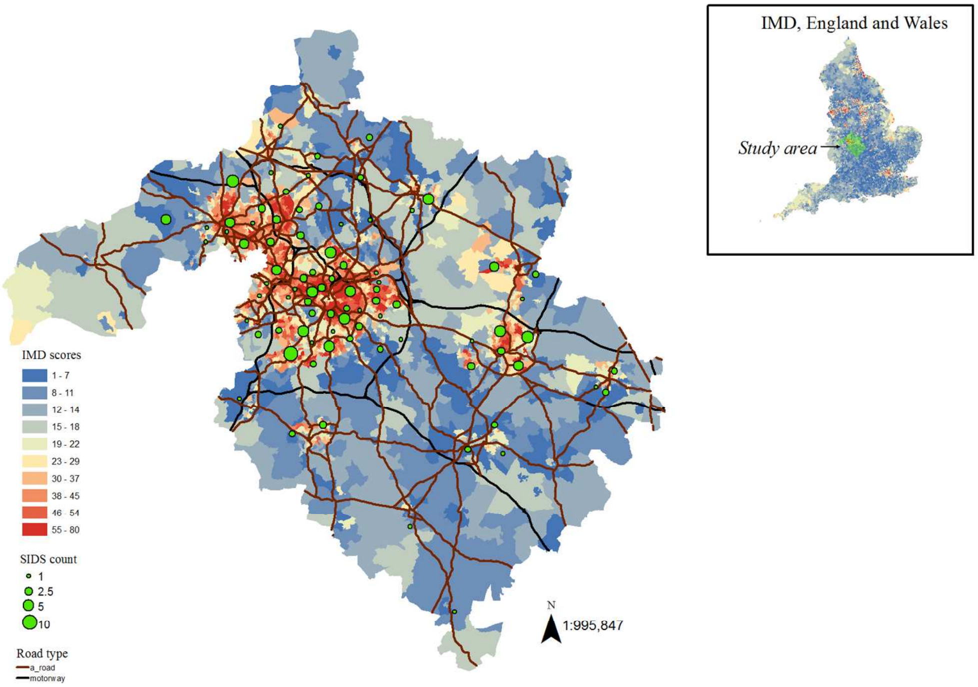

152x114mm (300 x 300 DPI)

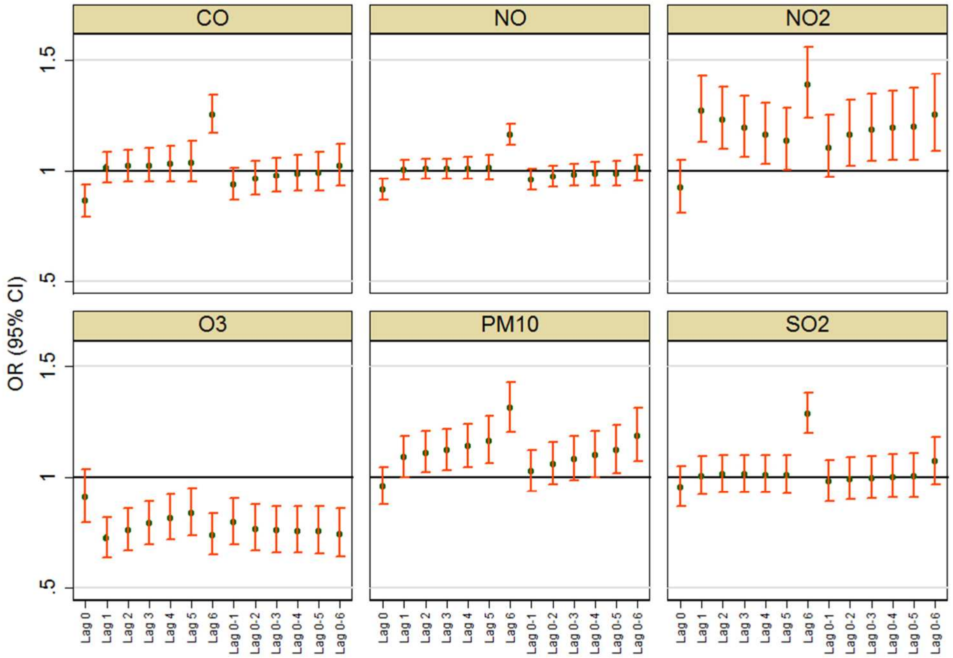

352x256mm (72 x 72 DPI)

STROBE Statement—Checklist of items that should be included in reports of ***case-control studies***

	Item No	Recommendation	Response
**Title and abstract**	1	(*a*) Indicate the study's design with a commonly used term in the title or the abstract	Case-crossover study included in title – Title page
		(*b*) Provide in the abstract an informative and balanced summary of what was done and what was found	Completed – including toning down the conclusion as per the associate editor's instruction page 2
**Introduction**			
Background/rationale	2	Explain the scientific background and rationale for the investigation being reported	Page 3
Objectives	3	State specific objectives, including any prespecified hypotheses	Page 3
**Methods**			
Study design	4	Present key elements of study design early in the paper	Page 4
Setting	5	Describe the setting, locations, and relevant dates, including periods of recruitment, exposure, follow-up, and data collection	Page 4
Participants	6	(*a*) Give the eligibility criteria, and the sources and methods of case ascertainment and control selection. Give the rationale for the choice of cases and controls	The study is a case-crossover study where effectively individuals act as their own controls<br>Page 4
		(*b*) For matched studies, give matching criteria and the number of controls per case	N/A
Variables	7	Clearly define all outcomes, exposures, predictors, potential confounders, and effect modifiers. Give diagnostic criteria, if applicable	Page 5
Data sources/ measurement	8*	For each variable of interest, give sources of data and details of methods of assessment (measurement). Describe comparability of assessment methods if there is more than one group	Page 4-6
Bias	9	Describe any efforts to address potential sources of bias	Pages 4-6
Study size	10	Explain how the study size was arrived at	Page 4
Quantitative variables	11	Explain how quantitative variables were handled in the analyses. If applicable, describe which groupings were chosen and why	Page 6
Statistical methods	12	(*a*) Describe all statistical methods, including those used to control for confounding	Page 4-6

For peer review only - http://bmjopen.bmj.com/site/about/guidelines.xhtml

For peer review only

		(*b*) Describe any methods used to examine subgroups and interactions	Page 6
		(*c*) Explain how missing data were addressed	Page 4
		(*d*) If applicable, explain how matching of cases and controls was addressed	N/A (Case-crossover study)
		(*e*) Describe any sensitivity analyses	Page 7
**Results**			
Participants	13*	(a) Report numbers of individuals at each stage of study—eg numbers potentially eligible, examined for eligibility, confirmed eligible, included in the study, completing follow-up, and analysed	Page 8
		(b) Give reasons for non-participation at each stage	Page 8
		(c) Consider use of a flow diagram	N/A
Descriptive data	14*	(a) Give characteristics of study participants (eg demographic, clinical, social) and information on exposures and potential confounders	Page 8
		(b) Indicate number of participants with missing data for each variable of interest	N/A
Outcome data	15*	Report numbers in each exposure category, or summary measures of exposure	Page 9-10
Main results	16	(*a*) Give unadjusted estimates and, if applicable, confounder-adjusted estimates and their precision (eg, 95% confidence interval). Make clear which confounders were adjusted for and why they were included	Page 9-10
		(*b*) Report category boundaries when continuous variables were categorized	N/A
		(*c*) If relevant, consider translating estimates of relative risk into absolute risk for a meaningful time period	N/A

For peer review only

Other analyses	17	Report other analyses done—eg analyses of subgroups and interactions, and sensitivity analyses	Page 11-12
**Discussion**			
Key results	18	Summarise key results with reference to study objectives	Page 13
Limitations	19	Discuss limitations of the study, taking into account sources of potential bias or imprecision. Discuss both direction and magnitude of any potential bias	Page 13
Interpretation	20	Give a cautious overall interpretation of results considering objectives, limitations, multiplicity of analyses, results from similar studies, and other relevant evidence	Page 14
Generalisability	21	Discuss the generalisability (external validity) of the study results	Page 15
**Other information**			
Funding	22	Give the source of funding and the role of the funders for the present study and, if applicable, for the original study on which the present article is based	Page 22

*Give information separately for cases and controls.

Note: An Explanation and Elaboration article discusses each checklist item and gives methodological background and published examples of transparent reporting. The STROBE checklist is best used in conjunction with this article (freely available on the Web sites of PLoS Medicine at http://www.plosmedicine.org/, Annals of Internal Medicine at http://www.annals.org/, and Epidemiology at http://www.epidem.com/). Information on the STROBE Initiative is available at http://www.strobe-statement.org.

For peer review only

For peer review only - http://bmjopen.bmj.com/site/about/guidelines.xhtml
